# Growing climatic sensitivity of U.S. agriculture linked to technological change and regional specialization

**DOI:** 10.1126/sciadv.aat4343

**Published:** 2018-12-12

**Authors:** Ariel Ortiz-Bobea, Erwin Knippenberg, Robert G. Chambers

**Affiliations:** 1 Charles H. Dyson School of Applied Economics and Management, Cornell University, Ithaca, NY 14853, USA.; 2 Cooper/Smith, Washington, DC 2006, USA.; 3 Department of Agricultural and Resource Economics, University of Maryland, College Park, MD 20742, USA.

## Abstract

A pressing question for climate change adaptation is whether ongoing transformations of the agricultural sector affect its ability to cope with climatic variations. We examine this question in the United States, where major increases in productivity have fueled most of agricultural production growth over the past half-century. To quantify the evolving climate sensitivity of the sector and identify its sources, we combine state-level measures of agricultural productivity with detailed climate data for 1960–2004. We find that agriculture is growing more sensitive to climate in Midwestern states for two distinct but compounding reasons: a rising climatic sensitivity of nonirrigated cereal and oilseed crops and a growing specialization in crop production. In contrast, other regions specialize in less climate-sensitive production such as irrigated specialty crops or livestock. Results suggest that reducing vulnerability to climate change should consider the role of policies in inducing regional specialization.

## Teaser

Core U.S. agricultural region has become more sensitive to climatic shocks due to changes in technology and specialization.

## INTRODUCTION

Sustaining growth in agricultural production is essential to meeting an ever-increasing global demand for food, fiber, and fuel (*1*–*7*). Continued productivity gains from technological progress are crucial to maintaining the global expansion of agricultural production (*8*, *9*), but efforts to make agriculture more productive must be balanced against efforts to increase its resilience to a changing climate (*10*–*12*). An important question is whether trade-offs exist between ongoing technological and structural transformations and agriculture’s adaptability to climatic hazards.

We consider this question for U.S. agriculture, which provides an ideal natural laboratory for examining the evolving linkages between productivity growth patterns and climatic sensitivity. The sector exhibits considerable regional variation in climate and production practices, while historic output growth has been primarily driven by productivity gains (*3*, *6*, *8*, *9*) and not by input growth. The ability to expand output without expanding its input base is usually attributed to technical change and distinguishes U.S. agriculture from most other industrial sectors where output growth is mainly attributable to input growth (*13*). Different drivers of that growth have been identified, with particular prominence given to public research and investment in U.S. agriculture (*4*, *6*, *8*, *9*).

To capture large-scale phenomena affecting a wide range of agricultural activities, we focus on agricultural total factor productivity (TFP). Agricultural TFP is defined as aggregate agricultural output divided by aggregate agricultural input. It is a standard economic measure of the sources of economic growth. The average rate of TFP growth measures the sector’s long-run ability to increase output without a corresponding increase in resource use. Because agricultural production is inherently stochastic, agricultural TFP growth is necessarily variable around that average. Changes in the relationship between the average growth rate and its variability can signal changes in agriculture’s adaptability to a changing production environment.

**Fig. 1:**
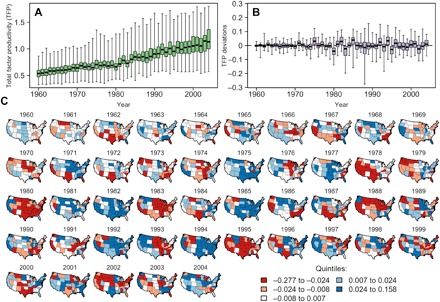
TFP distribution and deviations. (**A**) Distribution of TFP across the continental United States by year over 1960–2004. The box represents the first three quartiles, and whiskers extend to the extremes. TFP is normalized to 1 for Alabama in 1996. (**B**) Deviations in TFP from the trend based on a state-specific Hodrick-Prescott (HP) decomposition (see Materials and Methods). Boxes and whiskers are defined as for the level data. (**C**) Maps show yearly TFP deviations from state-level trend. The color scale corresponds to quintiles.

Figure 1 shows that the variation in agricultural TFP over time and across states over the continental U.S. has increased (Fig. 1A). TFP has also become more variable (Fig. 1B). Substantial TFP deviations from state trends appear increasingly common throughout the country, possibly reflecting the growing influence of climatic shocks (Fig. 1C). In this study, we use these TFP fluctuations and other aggregate indicators to examine the regional relationship between U.S. agricultural productivity and climatic variations and to disentangle the sources of its evolution. The study aims to characterize the evolution, if any, of the sensitivity of TFP to climatic shocks.

Our analysis relies on a panel model with fixed effects combined with a block-bootstrap procedure to analyze the sensitivity of U.S. agricultural TFP to climatic variations. As discussed in detail in Materials and Methods, we link official state-level TFP estimates from the U.S. Department of Agriculture (USDA) Economic Research Service (ERS) with detailed weather data from 1960 to 2004. We aggregate detailed 4-km gridded weather data to the state level based on 30-m cropland weights (fig. S1). We conduct the statistical analysis by USDA Climate Hub Region (fig. S2) in the lower 48 states and relate seasonal precipitation and nonlinear effects of temperature exposure (*14*) to detrended annual TFP.

## RESULTS

### The climatic sensitivity of agricultural TFP varies widely across the United States

**Fig. 2:**
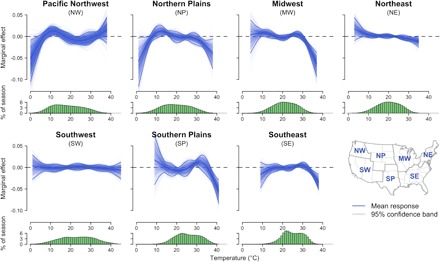
Productivity response to summer temperature by region. The overlay of blue response functions for each region corresponds to 1000 response functions derived from bootstrapped regressions in which years of data (1960–2004) were sampled with replacement. Mean and 95% confidence bands derived from the bootstrapped regressions are represented in darker lines. Functions were estimated separately for each USDA Climate Hub region based on state-level TFP and summer (June to August) weather data (see Materials and Methods). The green histograms below the response curves represent the percent of the time spent in each temperature bin during the summer season.

We find that TFP’s climatic sensitivity is regionally and seasonally heterogeneous. Exposure to relatively high summer temperature correlates with lower productivity in some regions, particularly in the Midwest (Fig. 2). We conduct a leave-one-year-out cross-validation to assess how weather variables help improve out-of-sample predictions in detrended TFP relative to a baseline model without weather variables. We find that summer (June to August) precipitation and temperature fluctuations explain much of the variation in detrended TFP in the Midwest [42.9% reduction in mean squared error (MSE)] and the Southeast (27.4%) (fig. S3). In contrast, summer weather explains a relatively small share of TFP variations in the Northeast (6.2%), the Northern Plains (5.6%), and the Southern Plains (5.4%) and virtually none in the Southwest and Northwest. Although summer weather explains much of the variation in detrended TFP, spring weather improves prediction in the Northern Plains (8.9% MSE reduction), whereas winter weather improves prediction in the Southwest (2.0%).

We find temperature to be the single best weather predictor of TFP variations. Specifically, the explanatory power of temperature exceeds that of precipitation alone in regions and seasons for which weather conditions help explain at least some of the variation in TFP, with the exception of the Southwest (fig. S3). Because their explanatory powers are not completely additive, precipitation and temperature variables may capture similar or correlated stressors reflecting drought conditions. Similarly, interactions between temperature and precipitation variables do not seem to improve explanatory power with a small exception in the Southeast.

These results are robust to alternative temperature and precipitation variable specifications (fig. S4). Increasing the flexibility of the spline for the temperature specification does not substantially alter explanatory power (fig. S4A). Similarly, modeling precipitation effects with a spline does not improve explanatory power relative to the basic quadratic representation that we favor (fig. S4B).

### The climatic sensitivity of agricultural TFP has evolved in certain regions, with hot summers becoming highly detrimental in the Midwest

A central question is whether climatic sensitivity of agricultural productivity has changed over time. We test for the stability of estimated weather parameters between the earlier (1960–1982) and later (1983–2004) periods. The statistical evidence indicates that sensitivity to summer temperature is different across these two periods in the Midwest (*P* = 0.032), whereas the sensitivity to precipitation has evolved in the Northwest (*P* = 0.026) and the Northeast (*P* = 0.049).

The evidence does not support evolving summer weather sensitivities in other regions over these two periods (table S1). The year 1983 was peculiar due to the simultaneous occurrence of a major drought and the introduction of the Payment-in-Kind Program, a major policy intervention aimed at reducing production. We conduct the same tests excluding 1983 and reach a similar conclusion (table S2).

To quantify the implications of the changes in the climatic sensitivity of agricultural productivity, we compute the effect of varying summer temperature changes on TFP for each region with models calibrated for both the earlier (1960–1982) and later (1983–2004) time periods. This exercise examines the effect of the changing sensitivities on TFP irrespective of any recent climatic trends. Because temperature anomalies are correlated with drought conditions that may be difficult to capture with seasonal precipitation, these scenarios should be interpreted as representing a broad range of environmental conditions associated with past temperature anomalies.

**Fig. 3:**
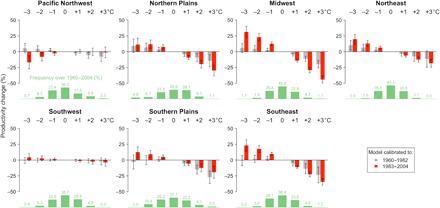
Predicted productivity changes from summer temperature change. Each panel represents the predicted changes in TFP corresponding to uniform changes of the entire summer temperature distribution. Predictions based on regressions calibrated with earlier data (1960–1982) are represented with gray bars, whereas predictions calibrated to more recent data (1983–2004) are represented with red bars. The error bars correspond to ±1 SD of the 1000 bootstrapped predictions. The green histogram represents the frequency of maximum summer temperature over the full sample period (1960–2004) and is presented for reference of the underlying distribution. The interval breaks fall within each full degree (e.g., the 2°C bin corresponds to summers between +1.5° and +2.5°C).

Figure 3 summarizes results for all regions and temperature scenarios. To illustrate, a 2°C warmer summer in the Midwest is associated with a 29.3% reduction in TFP in 1983–2004. In 1960–1982, identical summer weather conditions are associated with a much smaller 11.3% drop in TFP. This represents an important change in Midwestern climatic sensitivity. Currently, damaging summer conditions (+2°C) are relatively uncommon (6.4%) in this region (Fig. 3), but a 1°C uniform warming would more than quadruple their frequency to about 1 of every 4 years (29.2%). Under the latter scenario, the measured increased sensitivity could be especially problematic for Midwestern agricultural productivity performance. Note that these changes are in percentage terms, so they indicate that, while TFP is increasing over time, the climate-related variability in TFP is increasingly more pronounced. We conduct a similar analysis for uniform changes in summer precipitation and find these effects to be relatively small and stable over time (fig. S5).

While the changes in temperature effects on TFP between 1960–1982 and 1983–2004 are significant for the Midwest (*P* < 0.05), they appear largely insignificant for all other regions (*P* > 0.1), with the exception of the Southeast, where cooler summers appear somewhat more conducive to producing output (*P* < 0.1) (table S3). Although results are not statistically robust, warmer summers appear to have depressed output slightly less in the Western regions as well as in the Southern Plains. Last, because TFP measures can differ depending on the methods and data used in their construction (see Materials and Methods), we repeat our analysis for an alternative TFP dataset (*15*). Overall, results remain in close agreement (figs. S6 to S9).

### The climatic sensitivity of crop production has risen in the nonirrigated Eastern United States

Identifying the origins of changing TFP climatic sensitivities requires a more disaggregated analysis. TFP measures conflate all agricultural outputs. TFP estimates for subsectors, such as livestock or crops, are not practical because of the difficulty in ascribing input use across products. Instead, we analyze the behavior of output aggregates for crops and livestock. These aggregates represent more than 95% of the total national value produced over the study period. Although analyzing output sensitivities to climatic variations cannot account for the cost of farmer-controlled input responses, the exercise can elucidate evolving climatic sensitivities specific to agricultural subsectors in each region.

Crop production climatic sensitivity is heterogeneous across regions and seasons. Summer weather fluctuations explain a large share of detrended crop output variation in the Midwest (39.2% reduction in MSE relative to model without weather variables), the Southeast (32.7%), and the Northeast (26.1%) (fig. S10). Summer weather explains a smaller share of crop output variations in the Northern Plains (8.1%), the Southern Plains (8.0%), and the Northwest and the Southwest (negligible). Note, however, that weather conditions in the spring or the preceding winter improve prediction accuracy in the western regions. These findings are robust to alternative specifications of both the temperature and precipitation variables (fig. S11). Exposure to relatively high summer temperatures (>25°C) in various regions of the Eastern United States tends to reduce output (fig. S12). This finding correlates with microlevel crop-specific evidence (*14*, *16*, *17*). On the other hand, cooler conditions seem to reduce output in the Northwest. The lack of sensitivity to annual climatic variations in the West is likely linked to widespread and intensive use of irrigation in specialty crop production (fig. S13). Thus, these results may understate the West’s sensitivity to multiyear droughts not accounted for by this study.

Crop output has become increasingly sensitive to climate in parts of the United States, establishing a link with the change in TFP’s climatic sensitivity. Specifically, the sensitivity to summer temperature seems to differ between the early and later periods in the Midwest (*P* = 0.051) and the Southeast (*P* = 0.042), whereas that for precipitation appears to have evolved in the Northwest (*P* = 0.048). However, evidence of changing sensitivities of crop output in other regions is not found (table S4).

We also explore the effect of alternative warming and precipitation scenarios during the summer (fig. S13). The results suggest that higher temperatures only depressed output more in the later period in the Midwest (*P* < 0.05) (table S5). This growth in the climatic sensitivity of Midwestern crop output may partly explain the rising TFP sensitivity in this region (Fig. 3 and table S3). Although differences for other regions remain statistically insignificant, a trend appears to exist toward higher summer temperatures, reducing output more in the Northern Plains, the Northeast, and the Southeast (mostly nonirrigated). Higher summer temperatures appear to depress output less in the West and in the Southern Plains (mostly irrigated). This result for the Southern Plains may reflect its dependence on winter wheat, a cool-season crop that is not exposed to summer weather conditions (*16*, *17*).

### Livestock production remains relatively insensitive to climatic variations

TFP estimates also reflect the climatic sensitivity of livestock production, which is a large portion of the total U.S. agricultural output. An important finding is that climatic variations, regardless of the season, explain little to no variation in aggregate livestock production (fig. S15). This result is robust to alternative specifications of the temperature and precipitation variables (fig. S16), suggesting a relatively flat response function (fig. S17). We find no solid evidence of evolving sensitivities to summer climate variation over time (*P* > 0.05) (table S6). Thus, changes in livestock output under various scenarios of summer temperature and precipitation change appear small and mostly insignificant (fig. S18 and table S7). These results counter traditional intuition that livestock production, particularly dairy, is heat stress sensitive (*18*). Nonetheless, a supplementary and targeted analysis based on state-level monthly dairy output per cow over the same period confirms that temperature variations in both warm (July) and cold (January) months have a small or negligible influence on deviations in milk production (fig. S19).

One explanation is that livestock producers may have adopted effective strategies to cope with extreme heat. If these adjustments raise production costs substantially, then they could lower profitability, but our empirical analysis supports no clear association between aggregate input use and temperature fluctuations (fig. S20). This may happen because many agricultural input decisions are decided early in the season and thus remain fixed as relevant weather conditions unfold. It could also be that heat mitigation strategies are relatively simple and low cost (*19*) or mainly require input mix changes whose detection is elusive due to aggregation. Previous studies quantifying the effect of heat stress on livestock (*20*) suggest relatively modest impacts. Moreover, fuel and electricity costs, which may partly reflect the use of climate control systems, represent a relatively small fraction of production costs (table S8).

Although aggregate livestock production appears insensitive to climate, it is dependent on feed production, which is climate sensitive. In addition, some livestock operations produce field crops for on-farming feeding. This production and consumption cancels out and is not reflected directly in the productivity accounts but could enhance ultimate climate sensitivity of livestock. Major field crops such as climate-sensitive Midwestern maize and soybeans are primarily destined for animal consumption. We also find that hay production, a common source of animal feed throughout the country, exhibits temperature sensitivities much like crop output (fig. S21).

### Regional specialization in crop or livestock production alters TFP climatic sensitivity

Agricultural productivity may become more sensitive to climate because of the sector’s changing structure. For instance, a growing specialization into relatively more climate-sensitive production activities may increase TFP climatic sensitivity. Crop production appears more sensitive to climatic extremes than livestock production in various regions. This seems particularly true in the nonirrigated Eastern regions (see figs. S10 to S12 and S15 to S17). As a result, specialization in nonirrigated crop production increases TFP climatic sensitivity, while specialization in livestock production reduces it.

**Fig. 4:**
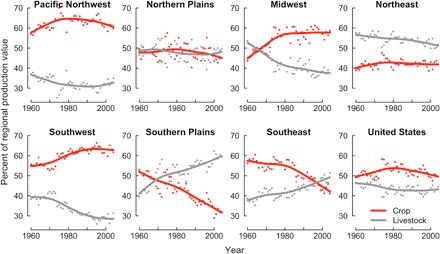
Regional specialization. Each panel represents the evolution of the share of each region’s production value in crops (red) and livestock (gray). Points indicate yearly observations, and lines correspond to a smooth spline trend line.

Figure 4 shows the evolution of the composition of the total output value for all regions. The Midwest has become increasingly specialized in nonirrigated cereal and oilseed production. The share of crop production in the total value of production in that region has increased from around 45% in 1960 to close to 60% in 2004. This increased specialization has increased the Midwest’s sensitivity to climatic hazards. This “specialization effect,” in turn, augments the already noted increasing climatic sensitivity of crop production (fig. S14). Multiple factors, including relative price changes and government policies, likely drive this increased specialization. This increased Midwestern specialization also paralleled the relocation of livestock production to neighboring regions (fig. S22) in the form of large-scale poultry and hog containment operations in the Southeast and of cattle feedlots in the Southern Plains.

Some regions became increasingly specialized in livestock production (Southern Plains and Southeast) or in the production of irrigated and specialty crops (Northwest and Southwest) that reduce overall climatic sensitivity (Fig. 4). For example, the growing specialization of the Southern Plains and the Southeast in livestock may explain why these regions did not experience increased TFP climatic sensitivity. On the other hand, the long-term success of the Southwest in reducing climatic sensitivity through irrigation relies on the enduring availability of Western water resources.

Some regions (the Northern Plains and the Northeast) maintained a relatively balanced growth between crops and livestock. The Northeast’s lasting specialization in livestock and dairy production makes it relatively insensitive to climatic variations despite its lack of widespread irrigation (figs. S3, S4, and S12).

## DISCUSSION

### Characterizing the overall regional climatic sensitivity of agricultural production

Previous work characterizing the climatic sensitivity of U.S. agricultural production has focused intensively on a relatively small number of commodities in a few regions. Consequently, it is well understood that nonirrigated, field crop yields in the Midwest and Eastern United States are sensitive to extreme weather (*14*, *16*), but field crops only account for approximately one-third of the national agricultural production value (*21*). The sensitivity of specialty crops or livestock in other regions remains poorly understood. Moreover, it remains unclear how climatic shocks to individual agricultural activities affect aggregate production performance at the regional or national levels. Because adaptive strategies to ameliorate climate resilience involve changes in both product mix and input mix (*22*–*24*), an accurate assessment of climatic effects on agriculture requires assessing aggregate performance.

A comprehensive evaluation of the evolving climatic sensitivity of the nation’s agricultural sector therefore must have two features. First, the benefits and costs of responding to climatic variations must be calculated. Coping with climatic extremes is costly; thus, the resulting effects on profitability must be understood. Second, all agricultural commodities and all regions must be considered. Agricultural production as well as strategies adopted to reduce climatic variability may vary widely across regions and globally. Moreover, national strategies for accommodating climate change could involve trading off increased climate sensitivity in some regions for decreased sensitivity elsewhere. Global strategies, in turn, may involve similar trade-offs across national boundaries. Thus, rising climatic sensitivity of agriculture in certain regions need not be undesirable if the associated gains in productivity lead to higher overall welfare and the proper mechanisms for sharing the risks across regions are established.

We examine these issues by relying on state-level measures of agricultural TFP. TFP is a keystone of economic growth accounting and is used to examine the sources of growth of industries or entire economies (*25*). TFP measures aggregate output per unit of aggregate input. The rate of TFP change thus measures output growth not explained by measured input growth and is the most commonly accepted measure of technological change (*26*). Because TFP incorporates all outputs and inputs into a single measure, it is better suited for describing aggregate phenomena than partial or single-input productivity measures such as crop yield or profit per acre that provide the basis for many studies of agriculture’s climatic sensitivity (*27*). Even under the most implausible assumptions, TFP is not a weighted average of partial productivity measures, such as crop yield, across crops. Hence, using these partial measures to make inferences about aggregate performance is not only erroneous but also potentially misleading. Proper measurement of aggregate performance requires proper accounting of all outputs and inputs. Existing TFP measures, which include material inputs and services in their construction, ignore natural inputs such as weather. Thus, to understand the effect of weather and climate change on agricultural performance, aggregate measures such as TFP must be combined with measurable weather variations. The agricultural TFP deviations we exploit in this study are ideal for examining the weather’s effect on agricultural productivity while accounting for farmer-controlled input adjustments.

### Sources of changing climatic sensitivity in U.S. agriculture

While agricultural production is inherently sensitive to climate, there are isolated indications that the climatic sensitivity of U.S. agriculture may be rising. For instance, Lobell *et al*. (*28*) find field-level evidence linking increasing average corn yields with greater sensitivity to drought in three Midwestern states. Roberts and Schlenker (*29*) find similar evidence pointing to decreasing heat tolerance of corn yields in Indiana since the 1960s. Elsewhere, Liang *et al*. (*30*) find that the link between national-level TFP changes and weather has intensified since the 1980s. Our study aims to fill the gap between field- and national-level evidence documenting these trends and to improve our understanding of its causes. Note that our study does not seek to attribute changes in long-term productivity trends to climatic trends.

Unraveling the sources of agriculture’s climatic sensitivity will improve our comprehension of its evolution. Our framework posits that the agricultural sector’s sensitivity to climatic shocks depends on both structural factors and the underlying technology. “Structure” reflects the sector’s composition. For example, by increasingly specializing in commodities with low climatic sensitivity, a region should reduce its overall climatic sensitivity. Structural transformations arise when farmers in a region alter the set of commodities they produce in response to changes in market prices and the policy context (e.g., subsidies). “Technology” reflects how inputs, including weather, are transformed into agricultural outputs. For example, agricultural technologies requiring increasing amounts of rain-fed moisture can make agricultural production increasingly climate sensitive. Technological transformations arise when farmers adopt new agricultural technologies that are often the result of private and public research and development (R&D).

Structure and technology interact to determine both regional and national sensitivity to climate change, and that interaction can be complex. For example, increased climate sensitivity in one region as a result of increased specialization could translate into decreased national agricultural sensitivity if that specialization decreases sensitivity in other regions. Similarly, irrigation-based crop production can appear less climate sensitive over shorter time horizons than rain-fed production, but if irrigation depletes available water sources, then it can increase climate sensitivity over long horizons.

We find that there is a growing climatic sensitivity in agricultural TFP that is concentrated in the core Midwest region. These findings are broadly consistent with national-level evidence (*30*). We isolate two explanatory factors. The first, consistent with structural change, is a growing Midwestern specialization in nonirrigated field crop production. The relative growth of a more climate-sensitive agricultural activity exacerbates the overall climatic sensitivity of this region. In the Southeast and the Southern Plains, we identify an opposing trend, in the form of increasing specialization in animal production. However, its impact on reducing overall climatic sensitivity appears too small to be detected. The second factor, consistent with technological change, is a rising sensitivity of nonirrigated field crop production to climatic factors in the Midwest. This result is consistent with microlevel evidence on corn (*28*).

A strength of our empirical strategy is the robust identification of the agricultural climatic sensitivity from annual weather and TFP deviations. However, this means that our findings only reflect the effects of random year-to-year weather fluctuations. Thus, the effect of prolonged multiyear droughts is not captured. This is important because these multiyear events would affect the availability of irrigation water in the West as well as the production of animal feed in the nonirrigated eastern parts of the country. Thus, we may understate the true long-term sensitivity of irrigated agriculture and animal production. In addition, our approach assesses the evolving role of weather in agricultural production implicitly by analyzing the effect of weather shocks on TFP. This precludes us from obtaining more nuanced insights about farmer responses to weather, which may be potentially elicited in a production-function framework in which weather is treated as an exogenous input alongside other farmer-controlled inputs in the derivation of TFP measures.

### Past and future agricultural adaptations to climate

Historically, climate has played a key role in U.S. agricultural development. Agriculture’s westward expansion was supported by persistent efforts to reduce its sensitivity to frigid and arid climatic conditions (*22*). It required advances in dryland farming and biological technologies (*23*), progress in groundwater extraction (*24*), and the evolution of the legal doctrine for appropriating scarce water resources (*31*). For example, extending wheat cultivation into colder Northern Plains climates required decades of trial and error to introduce and breed varieties adapted to what was then an inhospitable environment (*23*). Climate thus influenced the direction of R&D to enhance climate adaptation. Just as geographical expansion into different climate zones required adapting agricultural practices developed in other zones, sustained climate change may require altering both existing crop varieties and existing crop production mixes.

Technological progress in American agriculture endures (Fig. 1). Today, despite its relatively small contribution to gross domestic product, U.S. agriculture has accounted for 15% of total U.S. productivity growth since 1960 (*32*), and at times, agricultural productivity growth has averaged almost 10 times the economy-wide productivity growth rate. In addition, major structural transformations are still underway (Fig. 4). However, these rapid transformations have not reduced the sector’s sensitivity to climatic extremes. Rather, the evidence we report indicates an increased sectoral climatic sensitivity in some core areas. It is unclear from our findings whether the current trade-off between higher productivity and increased climatic sensitivity is socially desirable. However, our work suggests that the growing climatic sensitivity reported here will render the Midwest region more vulnerable in a warmer world. This increased sensitivity may become particularly problematic if, as other studies suggest, U.S. farmers fail to adapt to recent warming trends (*33*, *34*).

Climate change adaptation efforts must focus not only on technological adaptations needed to cope with climatic uncertainties but also on how agricultural policies and market realities affect the structure of agricultural production. Historically, U.S. commodity programs focused on Midwestern agriculture and were largely oriented toward supporting food grains, coarse grains, and oilseeds. The Renewable Fuel Standard and its associated ethanol mandates are more recent examples. Whether these policies played a decisive role in inducing the observed regional specialization that promoted that region’s increased vulnerability to climatic extremes remains unknown but an intriguingly plausible hypothesis. Understanding how government policies and market realities encourage structural adaptation in increasingly climate-vulnerable products is crucial to enhancing adaptation to a changing climate.

## MATERIALS AND METHODS

### Data sources

The USDA ERS data used in this study were obtained from ERS’s website (www.ers.usda.gov/data-products/agricultural-productivity-in-the-us/). The dataset provides state-level estimates of TFP as well as various output and input categories used in the construction of TFP estimates over the continental United States for 1960–2004. Unfortunately, the dataset has not been updated after 2004 because a critical source of labor information, the Farm Labor Survey, was discontinued. The methodology for estimating TFP is described on the ERS website (www.ers.usda.gov/data-products/agricultural-productivity-in-the-us/methods/) and is mostly based on (*35*). A critical review of the methods used in the construction of this dataset is provided in (*36*). The official state-level data used in this study were constructed following methods very similar to the national-level estimates used in (*30*). The main difference is that the state-level estimates account for interstate deliveries of output, which is not necessary in the national-level accounts. One practical question that arises in analyses involving broad aggregates such as crop production, livestock production, aggregate production, and aggregate input use is the treatment of commodities produced and used on a farm. As is consistent with economic theory, which treats such items as zero-valued netput, such items are treated as self-cancelling and do not enter into the accounts.

To verify the robustness of our findings, we replicate our analysis with TFP data from the International Science & Technology Practice & Policy (InSTePP) project at the University of Minnesota (*15*). This alternative dataset can be obtained from the project website (www.instepp.umn.edu/products/instepp-us-production-accounts-version-5-multifactor-productivity-index). Although the InSTePP data are available for a longer period (1949–2007), we restrict the supplementary analysis to the aforementioned period to facilitate comparisons. The main difference between the official and the InSTePP TFP datasets concerns the measurement of the capital stock and prices. For the official ERS data, the agricultural capital stock is measured using a perpetual inventory method. The InSTePP data measure capital stock using a physical inventory method. The perpetual inventory system, which is the more data intensive of the two, keeps track of stocks continuously and updates whenever investments are made. The physical inventory method instead relies on periodic counts.

Data on state-level milk production and hay yields were used for a supplementary analysis and were obtained from USDA National Agricultural Statistics Service (NASS). They can be accessed through the Quickstats online database (https://quickstats.nass.usda.gov).

Climate data were obtained from (*14*), which provides daily minimum and maximum temperature for each grid (4 km) over the continental United States for 1950–2005. This dataset is partly derived from monthly PRISM data from Oregon State University (*37*). To compute exposure to varying levels of temperature, we fitted a double-sine curve passing through the minimum and maximum temperature of each consecutive day at 15-min intervals. Exposures to each one-degree bin from −15° to 50°C were aggregated to the monthly level for each grid. Monthly gridded precipitation data were directly downloaded from the PRISM website (www.prism.oregonstate.edu). To obtain state-level climate data, we aggregate the gridded data using cropland weights based on USDA’s 30-m Cropland Data Layer for 2008–2014 (fig. S2). Although the total cropland area has fluctuated over the sample period, the spatial distribution of cropland areas has remained relatively stable within states over the past several decades (*38*). This weighting procedure captures the state-level climatic variations over agricultural land within the state.

### Statistical models

The HP filter is a widely used decomposition in macroeconomics to identify business cycles from time series economic data (*39*). The key parameter in the HP decomposition is the λ parameter, which should be chosen depending on the time resolution of the time series. We chose λ = 6.25, which is the usual practice for annual data (*40*). The econometric models are estimated based on detrended dependent variables using the HP filter. The HP filter operates as a flexible time trend, which avoids confounding cyclical downturns in productivity with climatic variations. We also consider alternative models with a Hub-specific quadratic time trend and non-detrended dependent variables (fig. S24) as with first-differenced dependent variables (fig. S25). We find similar findings in both cases.

We estimated the preferred econometric model separately for each USDA Climate Hub Region (fig. S2) using a panel estimator with state fixed effects, which can be represented as

$$y_{it}=\sum_{k=1}^{d}\gamma_{k}\underset{z_{it},k}{\underbrace{\sum_{h=\underline{h}}^{\bar{h}}T_{k}(h)[\Phi_{it}(h+1)-\Phi (h)]}}+\beta_{1}p_{it}+\beta_{2}p_{it}^{2}+\alpha_{i}+\epsilon_{it}$$

where *y*_*it*_ is the HP-filtered natural log of the outcome variable (e.g., TFP, crop, or livestock output) in state *i* and year *t*, $\sum_{k=1}^{d}\gamma_{k}z_{it,k}$ are the effects of temperature exposure, *p*_*it*_ is seasonal precipitation, α_*i*_ is a state dummy variable, and ε_*it*_ is an error term. Our approach is similar to that of Schlenker and Roberts (*14*) in that it allows nonlinear effects of temperature exposure over the season. More specifically, Φ_*it*_ (*h* + 1) – Φ_*it*_ (*h*) is the exposure to temperature bin *h* over the season, and *T*_*k*_(*h*) is the element in column *k* and row *h* of the basis matrix of a natural cubic spline with *d* degrees of freedom defined over temperature bins ($\underline{h},\ldots ,\bar{h}$). Unless otherwise noted, all models in the study rely on a natural spline specification with four degrees of freedom, which corresponds to 3 knots. All knots are equally spaced over the temperature support. In a sense, variable *z*_*it*,*k*_ is a locally weighted transformation of temperature bins, which assumes a smooth response to exposure to different temperature levels. We also considered polynomials and step function specifications of various degrees and widths and find the results to be fairly insensitive to the functional form. We show results based on a Chebyshev polynomial of degree 4 and a step function with 5°C steps in figs. S26 and S27. Note that we aggregate exposure at each tail of the full temperature distribution (−15° to 50°C) so that exposure to the extreme bins, $\underline{h}$ and $\bar{h}$, exceed at least 0.1% of the growing season during the sample period. Results are robust to higher thresholds (e.g., 0.5%; see fig. S28). The effects of precipitation are captured by the inclusion of linear and quadratic terms for seasonal precipitation. We consider various seasons in the study (figs. S3, S4, S10, S11, S15, and S16), including winter (preceding December, January, and February), spring (March, April, and May), summer (June, July, and August), fall (September, October, and November), and a longer warm period (April to September). We also explore splines with different degrees of flexibility for both temperature and precipitation (figs. S4, S11, and S16). We also consider whether weather variables have a lagged effect on TFP. However, we find that lagged weather variables have virtually no explanatory power on TFP fluctuations (fig. S29).

To represent the mean and variability of estimated effects and projections, we relied on a block-bootstrap procedure whereby we estimated the above model for each Climate Hub region 1000 times with data resampled by year. The resulting response functions are all represented on Fig. 2 and figs. S8, S12, S17, S19 to S21, and S24 to S28. The underlying variability in estimated impacts of uniform temperature and/or precipitation changes is represented with bars in Fig. 3 and figs. S5, S9, S14, S18, and S24 to S28. The mean and SDs necessary to test for differences in projected impacts for early (1960–1982) and late (1982/1983–2004) periods in tables S3, S5, and S7 were based on the bootstrapped projections.

## Supplementary Material

http://advances.sciencemag.org/cgi/content/full/4/12/eaat4343/DC1

## SUPPLEMENTARY MATERIALS

Supplementary material for this article is available at http://advances.sciencemag.org/cgi/content/full/4/12/eaat4343/DC1 Fig. S1. Map of cropland weights. Fig. S2. Map of USDA Climate Hub regions. Fig. S3. Average reduction in MSE of out-of-sample predictions in TFP relative to a model without weather variables (1960–2004). Fig. S4. TFP predictability under varying flexibilities for temperature and precipitation variables. Fig. S5. Predicted productivity changes from summer precipitation change. Fig. S6. Average reduction in MSE in TFP relative to a model without weather variables (1960–2004) based on alternative production dataset. Fig. S7. Average reduction in MSE in TFP relative to a model without weather variables (1960–2004) based on alternative production dataset. Fig. S8. Productivity response to summer temperature by region based on alternative production dataset. Fig. S9. Predicted productivity changes based on alternative TFP dataset. Fig. S10. Average reduction in MSE of out-of-sample predictions in crop output relative to a model without weather variables (1960–2004). Fig. S11. Crop output predictability under varying flexibilities for temperature and precipitation variables. Fig. S12. Crop output response to summer temperature by region. Fig. S13. Estimated water use for crop and livestock production in the United States. Fig. S14. Predicted crop output changes. Fig. S15. Average reduction in MSE of out-of-sample predictions in livestock output relative to a model without weather variables (1960–2004). Fig. S16. Crop output predictability under varying flexibilities for temperature and precipitation variables. Fig. S17. Livestock output response to summer temperature by region. Fig. S18. Predicted livestock output changes. Fig. S19. Milk production per cow response to monthly temperature by region. Fig. S20. Aggregate input response to summer temperature by region. Fig. S21. Hay yield response to summer temperature by region. Fig. S22. Growth of regional production value. Fig. S23. Contribution to national production. Fig. S24. Productivity response to summer temperature change based on a quadratic time trend. Fig. S25. Productivity response to summer temperature change based on first differences. Fig. S26. Productivity response to summer temperature change based on a Chebyshev polynomial of degree 4. Fig. S27. Productivity response to summer temperature change based on a step function with 5°C steps. Fig. S28. Productivity response to summer temperature change based on a higher tail aggregation threshold of 0.5%. Fig. S29. Average reduction in MSE in TFP relative to a model without weather variables (1960–2004) for models with lagged weather variables. Table S1. Test results for climate parameter stability in TFP regressions. Table S2. Test results for climate parameter stability in TFP regressions excluding 1983. Table S3. Estimates and *P* values for test of differences in impact on TFP of varying temperature scenarios. Table S4. Test results for climate parameter stability in crop output regressions. Table S5. Estimates and *P* values for test of differences in impact on crop output of varying temperature scenarios. Table S6. Test results for climate parameter stability in livestock output regressions. Table S7. Estimates and *P* values for test of differences in impact on livestock output of varying temperature scenarios. Table S8. Decomposition of production costs by livestock output category, farm resource region, and over time (in %).
