# Supplementary material for: Growing climatic sensitivity of U.S. agriculture linked to technological change and regional specialization
Source: Sci Adv. 2018 Dec 12;4(12):eaat4343. doi: 10.1126/sciadv.aat4343 (PMC6291312; doi:10.1126/sciadv.aat4343)
Supplement: http://advances.sciencemag.org/cgi/content/full/4/12/eaat4343/DC1 [file supp_4_12_eaat4343__index.html]

Science Advances | Science Advances

## Supplementary Materials

**This PDF file includes:**

- Fig. S1. Map of cropland weights.
- Fig. S2. Map of USDA Climate Hub regions.
- Fig. S3. Average reduction in MSE of out-of-sample predictions in TFP relative to a model without weather variables (1960–2004).
- Fig. S4. TFP predictability under varying flexibilities for temperature and precipitation variables.
- Fig. S5. Predicted productivity changes from summer precipitation change.
- Fig. S6. Average reduction in MSE in TFP relative to a model without weather variables (1960–2004) based on alternative production dataset.
- Fig. S7. Average reduction in MSE in TFP relative to a model without weather variables (1960–2004) based on alternative production dataset.
- Fig. S8. Productivity response to summer temperature by region based on alternative production dataset.
- Fig. S9. Predicted productivity changes based on alternative TFP dataset.
- Fig. S10. Average reduction in MSE of out-of-sample predictions in crop output relative to a model without weather variables (1960–2004).
- Fig. S11. Crop output predictability under varying flexibilities for temperature and precipitation variables.
- Fig. S12. Crop output response to summer temperature by region.
- Fig. S13. Estimated water use for crop and livestock production in the United States.
- Fig. S14. Predicted crop output changes.
- Fig. S15. Average reduction in MSE of out-of-sample predictions in livestock output relative to a model without weather variables (1960–2004).
- Fig. S16. Crop output predictability under varying flexibilities for temperature and precipitation variables.
- Fig. S17. Livestock output response to summer temperature by region.
- Fig. S18. Predicted livestock output changes.
- Fig. S19. Milk production per cow response to monthly temperature by region.
- Fig. S20. Aggregate input response to summer temperature by region.
- Fig. S21. Hay yield response to summer temperature by region.
- Fig. S22. Growth of regional production value.
- Fig. S23. Contribution to national production.
- Fig. S24. Productivity response to summer temperature change based on a quadratic time trend.
- Fig. S25. Productivity response to summer temperature change based on first differences.
- Fig. S26. Productivity response to summer temperature change based on a Chebyshev polynomial of degree 4.
- Fig. S27. Productivity response to summer temperature change based on a step function with 5°C steps.
- Fig. S28. Productivity response to summer temperature change based on a higher tail aggregation threshold of 0.5%.
- Fig. S29. Average reduction in MSE in TFP relative to a model without weather variables (1960–2004) for models with lagged weather variables.
- Table S1. Test results for climate parameter stability in TFP regressions.
- Table S2. Test results for climate parameter stability in TFP regressions excluding 1983.
- Table S3. Estimates and *P* values for test of differences in impact on TFP of varying temperature scenarios.
- Table S4. Test results for climate parameter stability in crop output regressions.
- Table S5. Estimates and *P* values for test of differences in impact on crop output of varying temperature scenarios.
- Table S6. Test results for climate parameter stability in livestock output regressions.
- Table S7. Estimates and *P* values for test of differences in impact on livestock output of varying temperature scenarios.
- Table S8. Decomposition of production costs by livestock output category, farm resource region, and over time (in %).

Download PDF

**Files in this Data Supplement:**

- Adobe PDF - aat4343\_SM.pdf
