# Supplementary material for: Growing climatic sensitivity of U.S. agriculture linked to technological change and regional specialization
Source: Sci Adv. 2018 Dec 12;4(12):eaat4343. doi: 10.1126/sciadv.aat4343 (PMC6291312; doi:10.1126/sciadv.aat4343)
Supplement: http://advances.sciencemag.org/cgi/content/full/4/12/eaat4343/DC1 [file aat4343_SM.pdf]

Science Advances
AAAS

advances.sciencemag.org/cgi/content/full/4/12/eaat4343/DC1

Supplementary Materials for

Growing climatic sensitivity of U.S. agriculture linked to technological change and regional specialization

Ariel Ortiz-Bobea*, Erwin Knippenberg, Robert G. Chambers

*Corresponding author. Email: ao332@cornell.edu

Published 12 December 2018, *Sci. Adv.* **4**, eaat4343 (2018)
DOI: 10.1126/sciadv.aat4343

This PDF file includes:

Fig. S1. Map of cropland weights.
Fig. S2. Map of USDA Climate Hub regions.
Fig. S3. Average reduction in MSE of out-of-sample predictions in TFP relative to a model without weather variables (1960–2004).
Fig. S4. TFP predictability under varying flexibilities for temperature and precipitation variables.
Fig. S5. Predicted productivity changes from summer precipitation change.
Fig. S6. Average reduction in MSE in TFP relative to a model without weather variables (1960–2004) based on alternative production dataset.
Fig. S7. Average reduction in MSE in TFP relative to a model without weather variables (1960–2004) based on alternative production dataset.
Fig. S8. Productivity response to summer temperature by region based on alternative production dataset.
Fig. S9. Predicted productivity changes based on alternative TFP dataset.
Fig. S10. Average reduction in MSE of out-of-sample predictions in crop output relative to a model without weather variables (1960–2004).
Fig. S11. Crop output predictability under varying flexibilities for temperature and precipitation variables.
Fig. S12. Crop output response to summer temperature by region.
Fig. S13. Estimated water use for crop and livestock production in the United States.
Fig. S14. Predicted crop output changes.
Fig. S15. Average reduction in MSE of out-of-sample predictions in livestock output relative to a model without weather variables (1960–2004).
Fig. S16. Crop output predictability under varying flexibilities for temperature and precipitation variables.
Fig. S17. Livestock output response to summer temperature by region.
Fig. S18. Predicted livestock output changes.
Fig. S19. Milk production per cow response to monthly temperature by region.
Fig. S20. Aggregate input response to summer temperature by region.

Fig. S21. Hay yield response to summer temperature by region.
Fig. S22. Growth of regional production value.
Fig. S23. Contribution to national production.
Fig. S24. Productivity response to summer temperature change based on a quadratic time trend.
Fig. S25. Productivity response to summer temperature change based on first differences.
Fig. S26. Productivity response to summer temperature change based on a Chebyshev polynomial of degree 4.
Fig. S27. Productivity response to summer temperature change based on a step function with 5°C steps.
Fig. S28. Productivity response to summer temperature change based on a higher tail aggregation threshold of 0.5%.
Fig. S29. Average reduction in MSE in TFP relative to a model without weather variables (1960–2004) for models with lagged weather variables.
Table S1. Test results for climate parameter stability in TFP regressions.
Table S2. Test results for climate parameter stability in TFP regressions excluding 1983.
Table S3. Estimates and *P* values for test of differences in impact on TFP of varying temperature scenarios.
Table S4. Test results for climate parameter stability in crop output regressions.
Table S5. Estimates and *P* values for test of differences in impact on crop output of varying temperature scenarios.
Table S6. Test results for climate parameter stability in livestock output regressions.
Table S7. Estimates and *P* values for test of differences in impact on livestock output of varying temperature scenarios.
Table S8. Decomposition of production costs by livestock output category, farm resource region, and over time (in %).

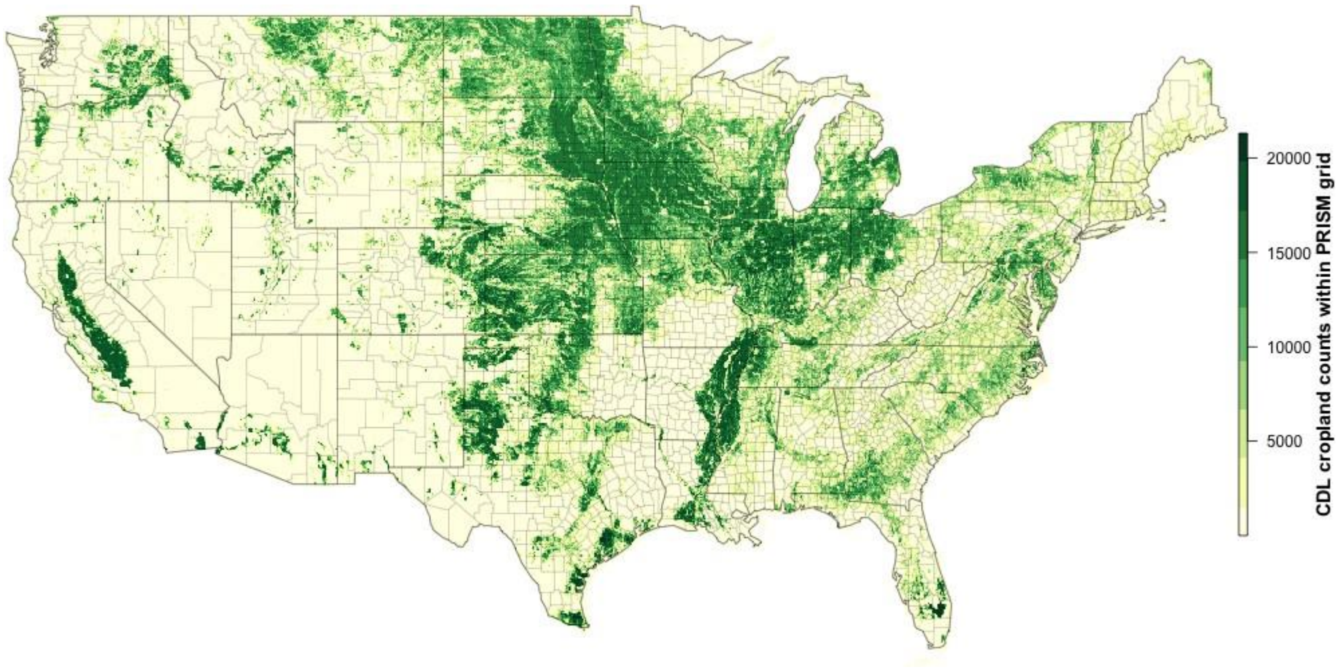

Fig. S1. Map of cropland weights. The cropland weights are used to aggregate the 4-km gridded weather data to the state level, as a way to avoid using weather data grids that do not have agricultural-related activities. We rely on the USDA Crop Data Layer (CDL), which is a 30-m land cover data for the continental United States. We compute the cropland weights by computing the total number of crop pixels falling within each 4-km grid of the PRISM/SR grid for each year in 2008-2014, which we then average over time. The average cropland count is depicted in the above figure. CDL data was obtained from the following webpage: https://www.nass.usda.gov/Research and Science/Cropland/Release/index.php

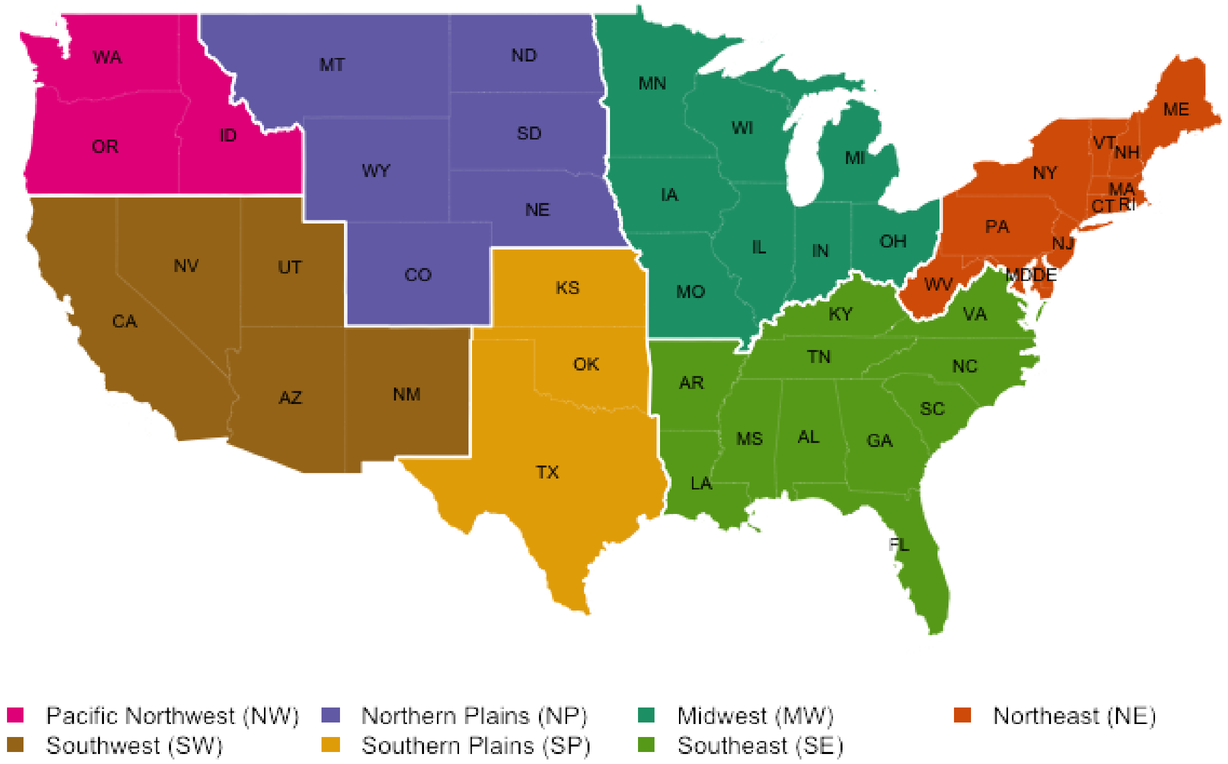

Fig. S2. Map of USDA Climate Hub regions. Climate hubs provide administratively relevant and agro-climatically consistent units of analysis. We conduct our analysis at the yearly and state level by climate hub region. More details regarding the Climate Hubs can be obtained at: https://www.climatehubs.oce.usda.gov

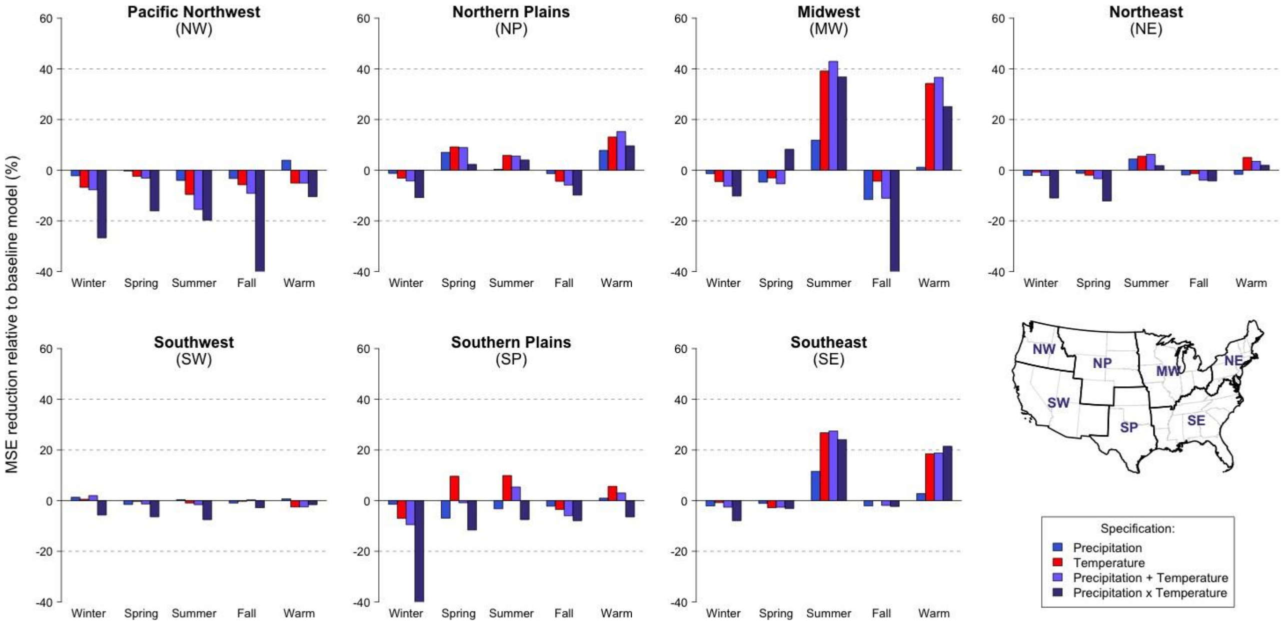

Fig. S3. Average reduction in MSE of out-of-sample predictions in TFP relative to a model without weather variables (1960–2004). MSE is computed as the average MSE of a leave-one-out cross-validation in which observations are excluded one year at a time for each climate hub region. The "Precipitation" model incorporates both linear and quadratic terms for total precipitation over the relevant period. The "Temperature" specification models temperature exposure to varying temperature levels with a natural spline with 4 degrees of freedom (3 knots). The "Precipitation + Temperature" model incorporates both types of variables with- out interactions. The "Precipitation × Temperature" model augments the previous model with interaction terms between precipitation and temperature variables. The seasons con- sidered include: Winter (Dec, Jan, Feb), Spring (Mar, Apr, May), Summer (Jun, Jul, Aug), Fall (Sep, Oct, Nov) and a longer Warm period (Apr-Sep). In some seasons, weather does not explain TFP variations, which results in negative MSE reductions relative to a model without weather variables.

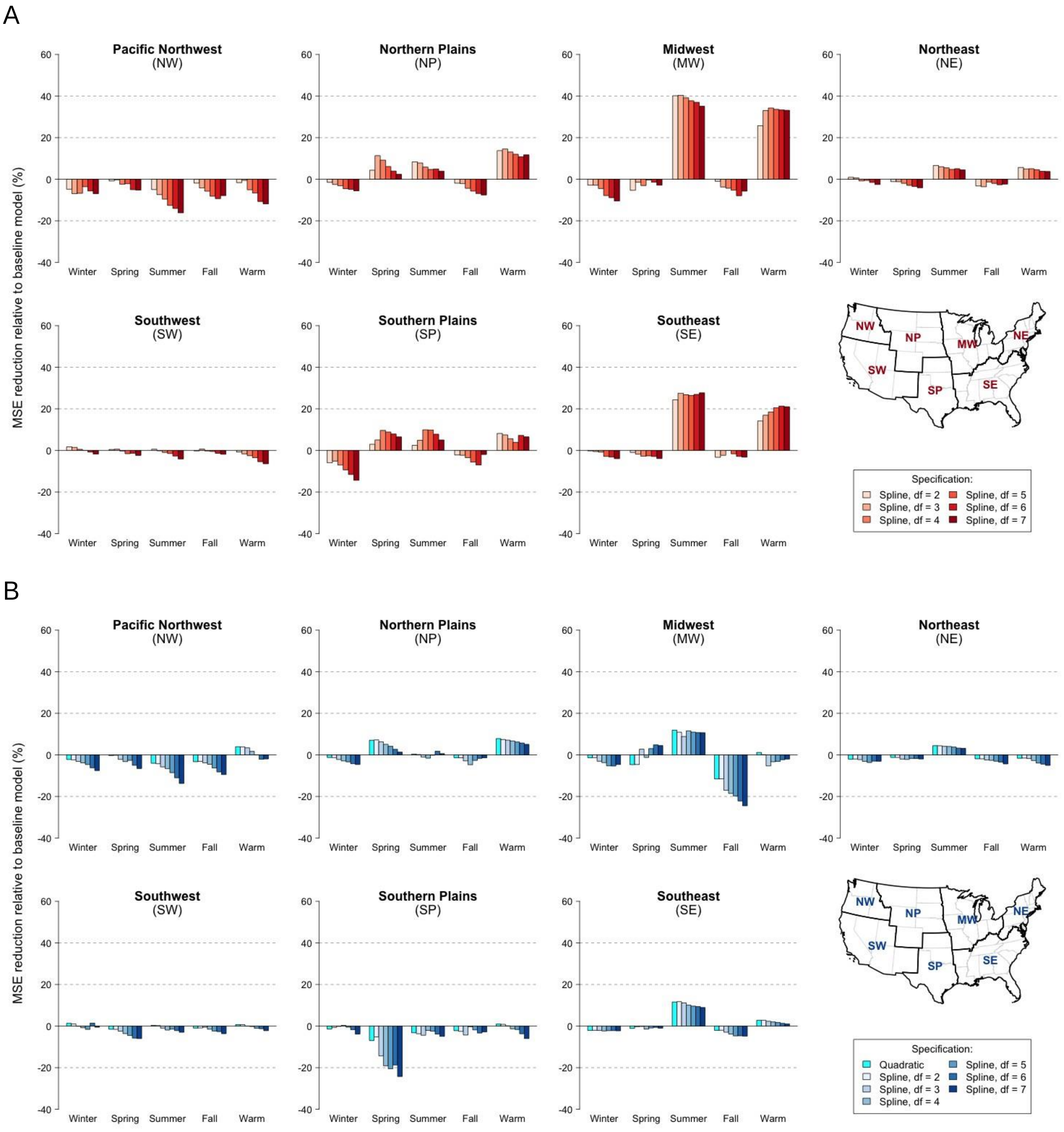

Fig. S4. TFP predictability under varying flexibilities for temperature and precipitation variables. Average reduction in Mean Squared Error (MSE) of out-of-sample predictions in Total Factor Productivity (TFP) relative to a model without weather variables (1960-2004) for varying levels of flexibility of (A) temperature and (B) precipitation response functions. The "quadratic" specification in panel B includes linear and quadratic terms for total precipitation for the relevant period. Details about the spline specifications and how the MSE reductions are computed are provided in *Methods*.

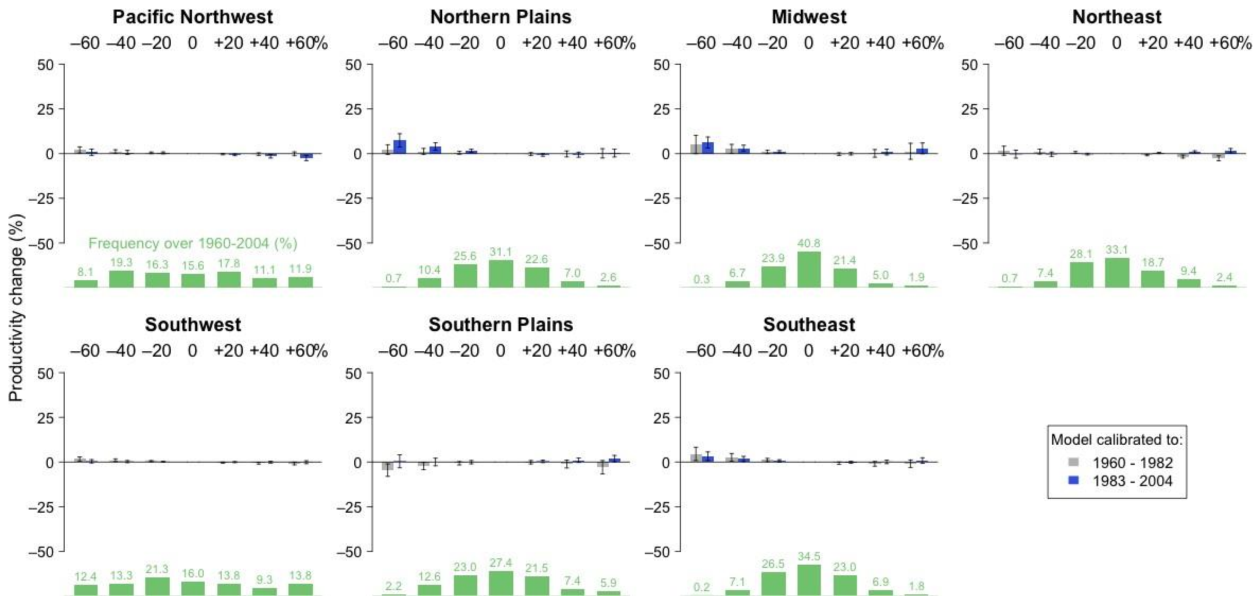

Fig. S5. Predicted productivity changes from summer precipitation change. The figure represents the same analysis shown in Fig. 3 in the paper but applied to uniform changes in precipitation.

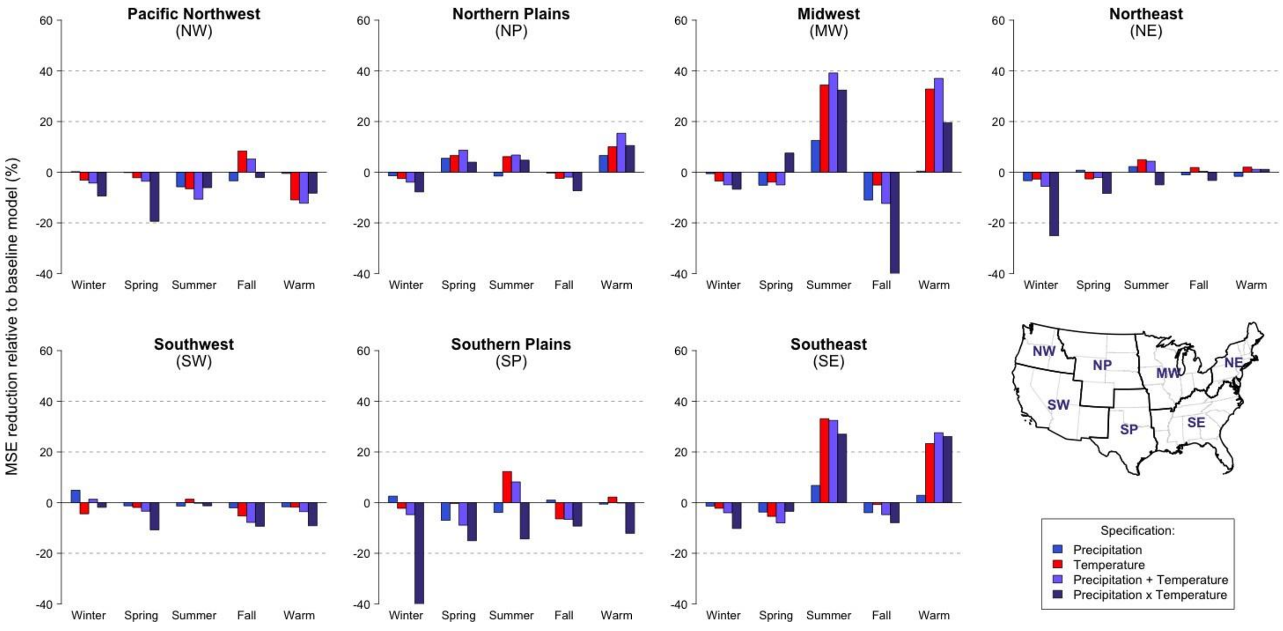

Fig. S6. Average reduction in MSE in TFP relative to a model without weather variables (1960–2004) based on alternative production dataset. The figure represents the same analysis shown in fig. S3 but relies on an alternative TFP dataset (Pardey et al. 2014).

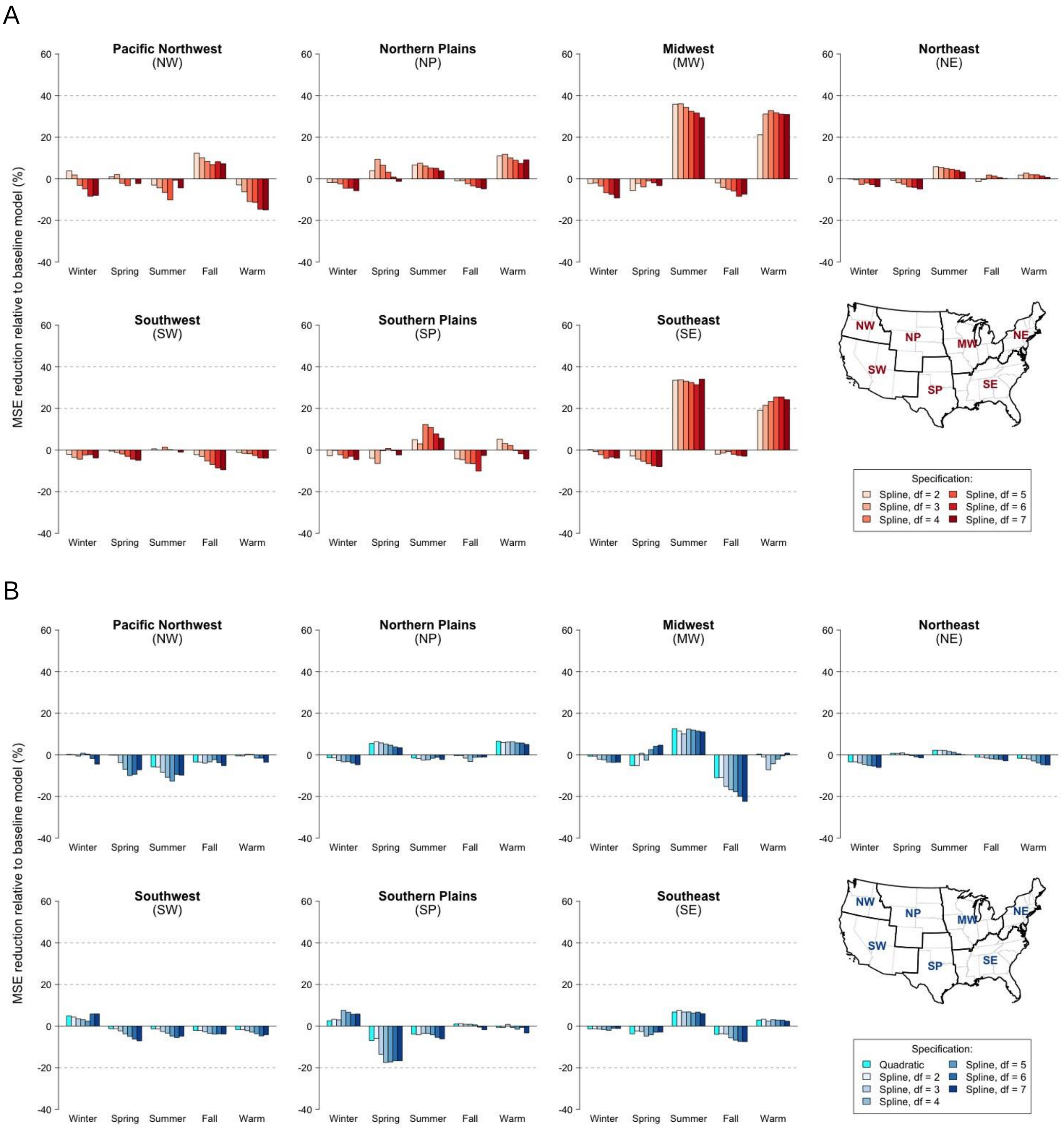

Fig. S7. Average reduction in MSE in TFP relative to a model without weather variables (1960–2004) based on alternative production dataset. The figure represents the same analysis shown in fig. S4 but relies on an alternative TFP dataset (Pardey et al. 2014).

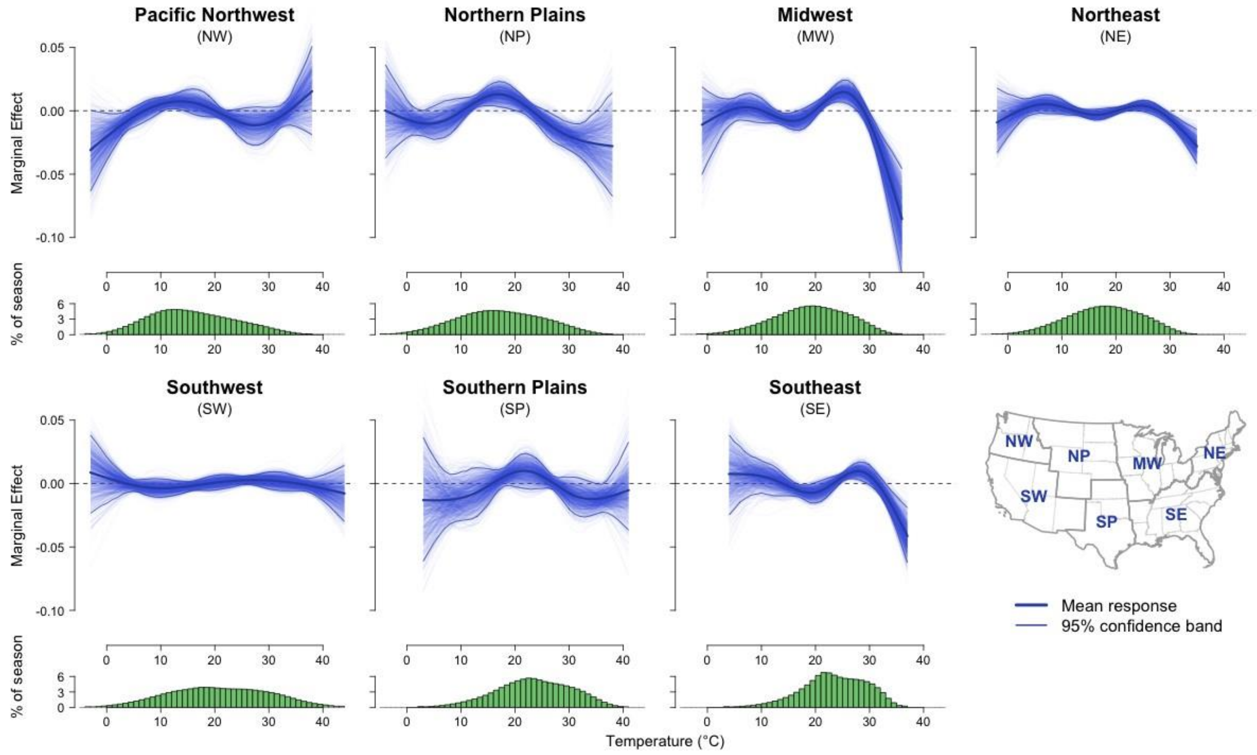

Fig. S8. Productivity response to summer temperature by region based on alternative production dataset. The figure represents the same analysis shown in Fig. 2 in the paper but relies on an alternative TFP dataset (Pardey et al. 2014). The overlay of blue response functions for each region corresponds to 1,000 response functions derived from bootstrapped regressions in which years of data (1960-2004) were sampled with replacement. Mean and 95% confidence bands derived from the bootstrapped regressions are represented in darker lines. Functions were estimated separately for each USDA Climate Hub region based on state-level TFP and summer (June-August) weather data (see *Methods*). The green histograms below the response curves represent the percent of the time spent in each temperature bin during the summer.

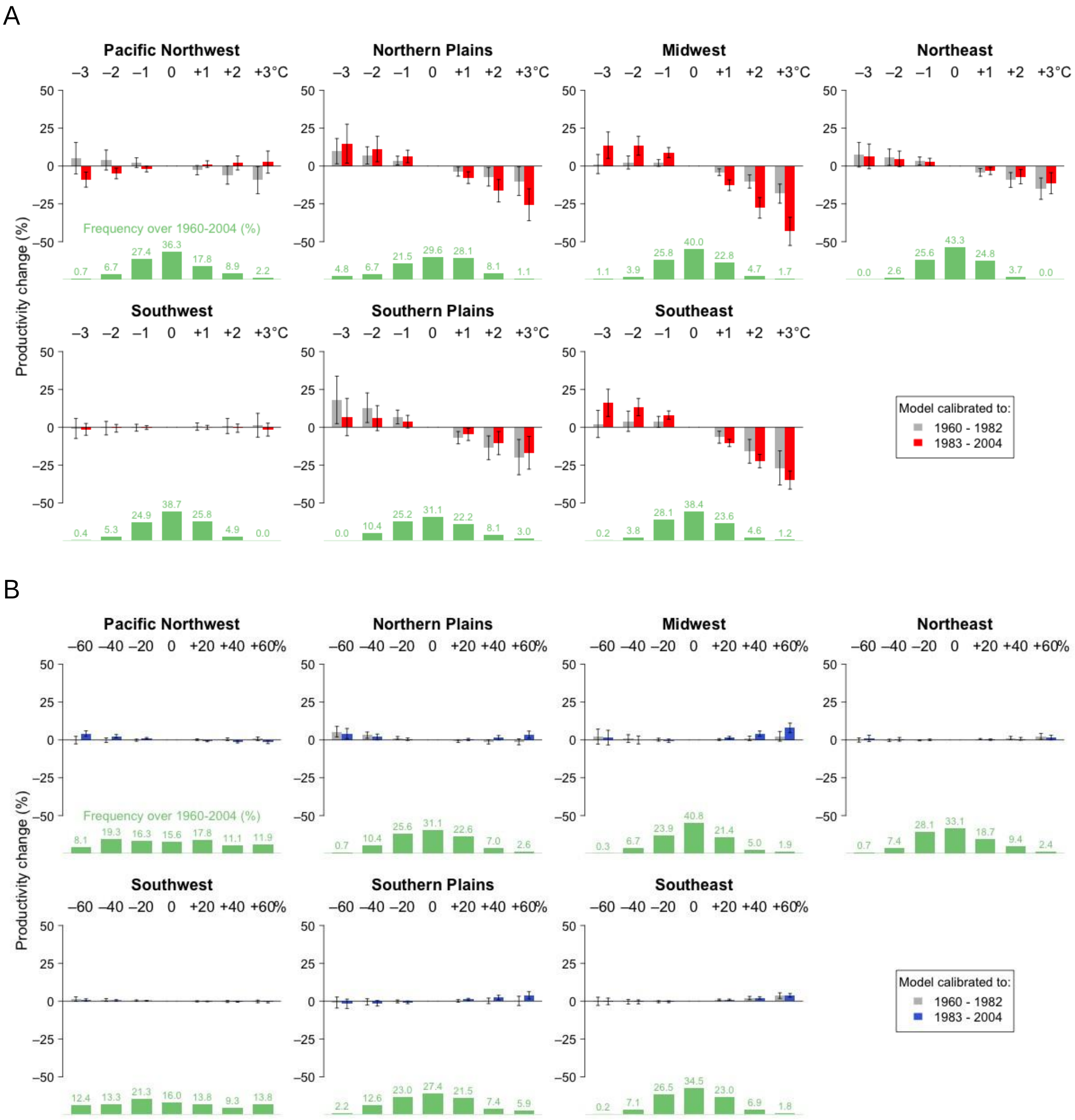

Fig. S9. Predicted productivity changes based on alternative TFP dataset. (**A** and **B**) The figure represents the same analysis shown in Fig. 3 and fig. S5 but relies on an alternative TFP dataset (Pardey et al. 2014).

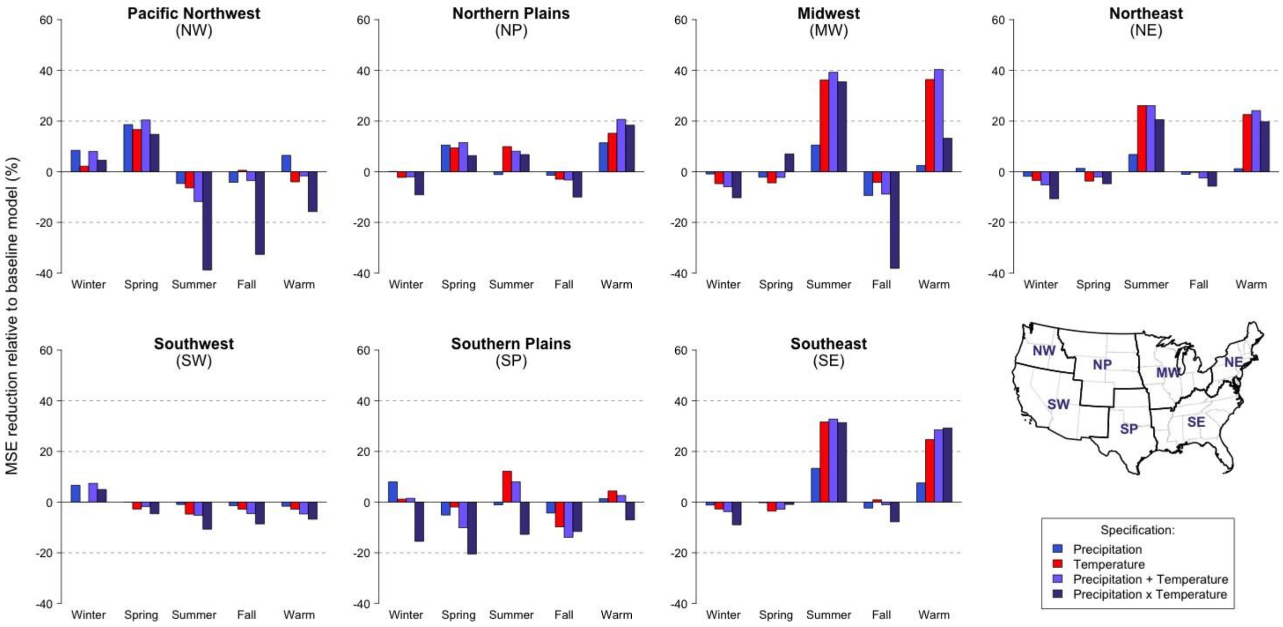

Fig. S10. Average reduction in MSE of out-of-sample predictions in crop output relative to a model without weather variables (1960–2004). This figure is analogous to fig. S3 but applied to crop output.

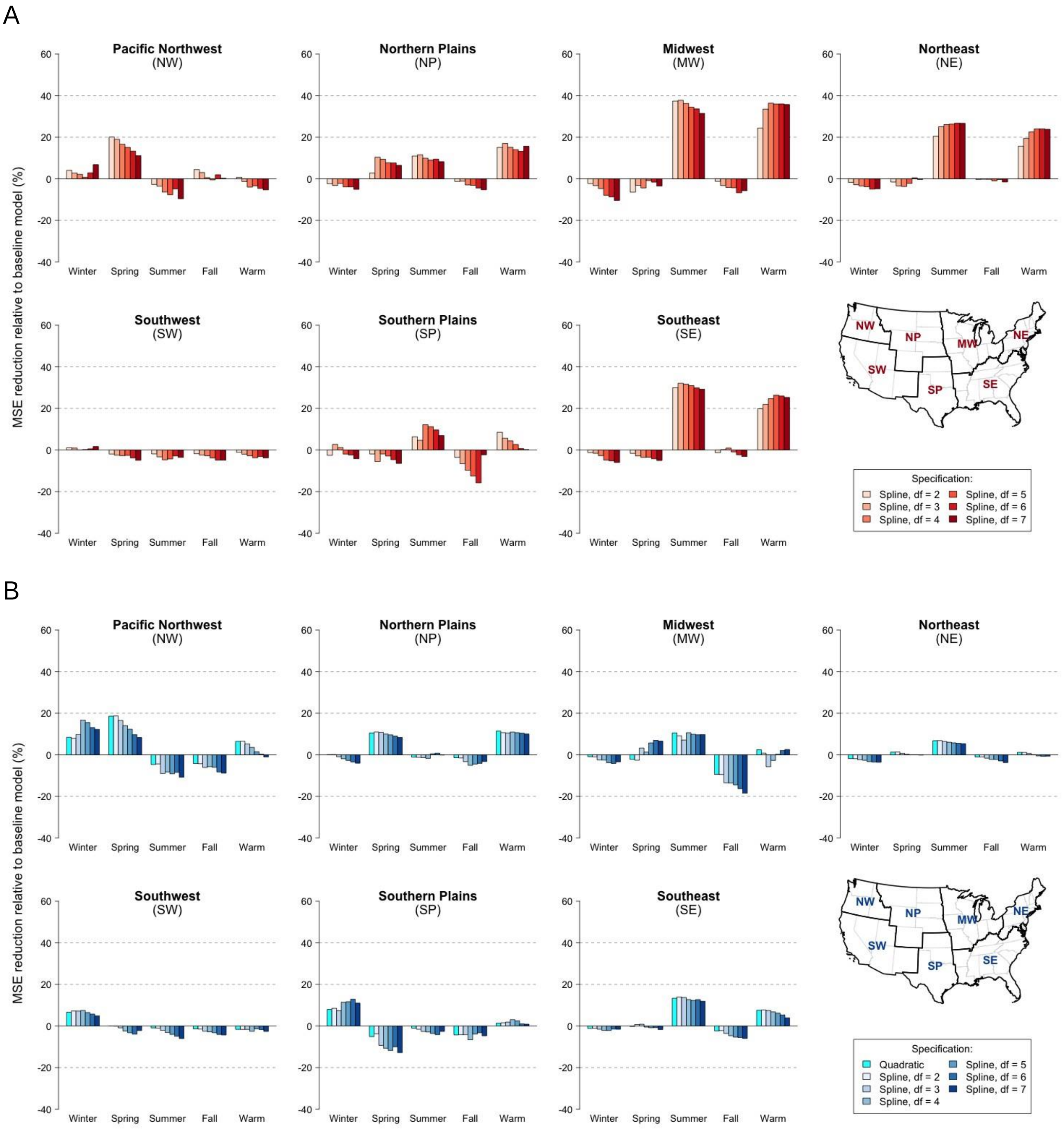

Fig. S11. Crop output predictability under varying flexibilities for temperature and precipitation variables. Average reduction in Mean Squared Error (MSE) of out-of-sample predictions in Crop Output relative to a model without weather variables (1960- 2004) for varying levels of flexibility of (**A**) temperature and (**B**) precipitation response functions. This figure is analogous to fig. S4 but applied to crop output.

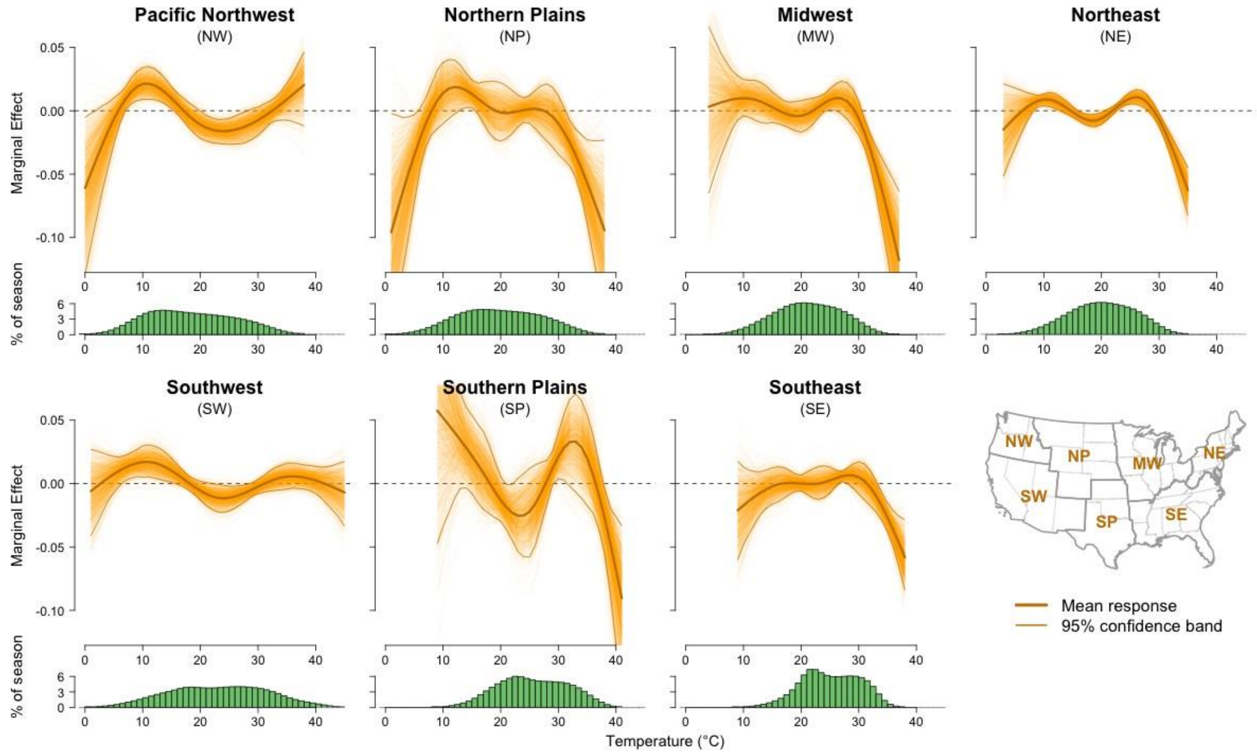

Fig. S12. Crop output response to summer temperature by region. The overlay of gold response functions for each region corresponds to 1,000 response functions derived from bootstrapped regressions in which years of data (1960-2004) were sampled with replacement. Mean and 95% confidence intervals are represented in darker lines. Functions were estimated separately for each USDA climate hub region based on state-level crop output and summer (June-August) weather data. The green histograms below the response curves represent the percent of the time spent in each temperature bin during the summer.

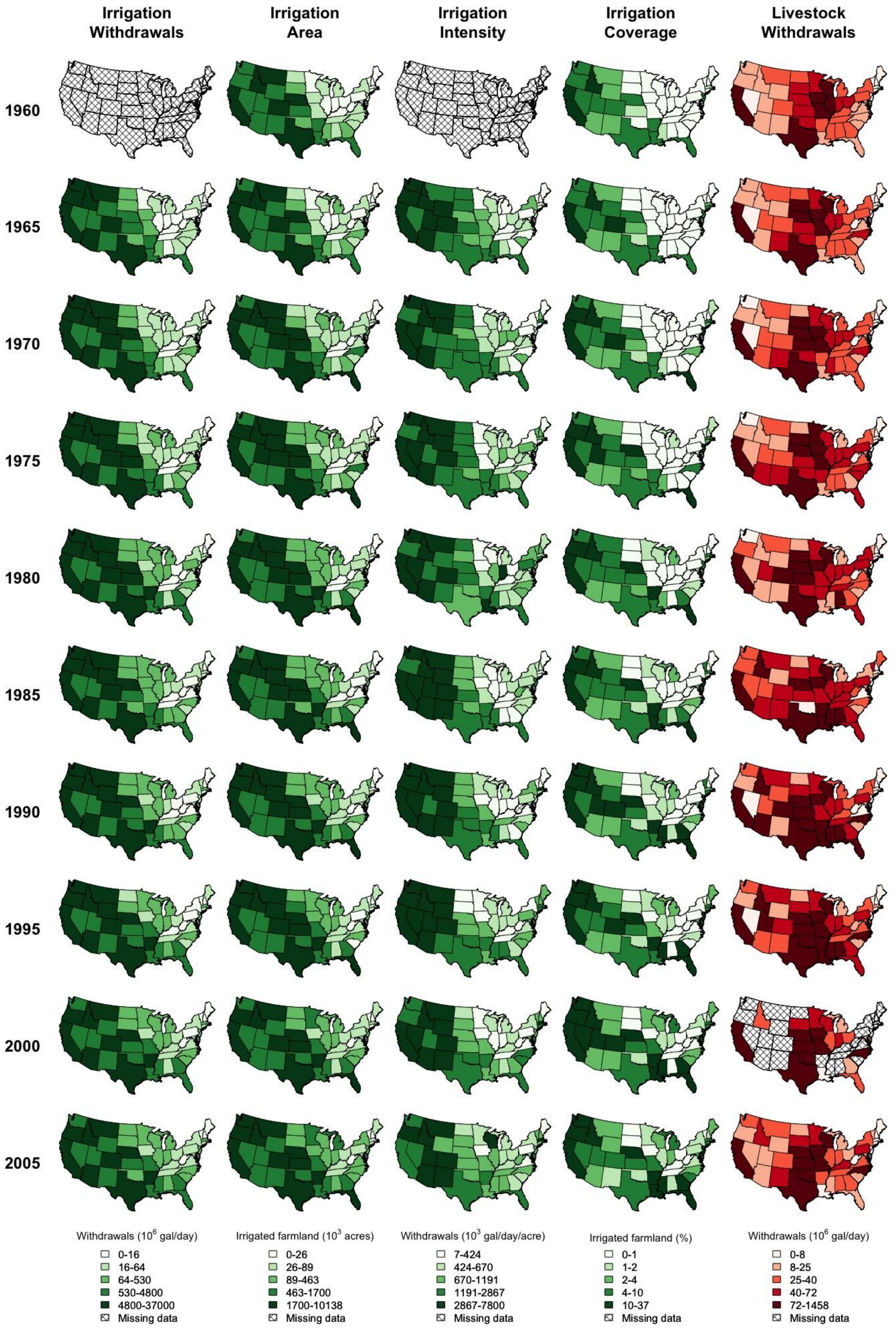

Fig. S13. Estimated water use for crop and livestock production in the United States. Irrigation withdrawals represent the total (surface and groundwater) withdrawals used for irrigation. Irrigation area corresponds acreage of irrigated farmland. Irrigation intensity reflects withdrawals per acre of irrigated farmland. Irrigation coverage reflects the share of farmland that is irrigated. Data Sources: United States Geological Survey for water statistics (https://water.usgs.gov/watuse/data/) and USDA Census of Agriculture for total farmland area.

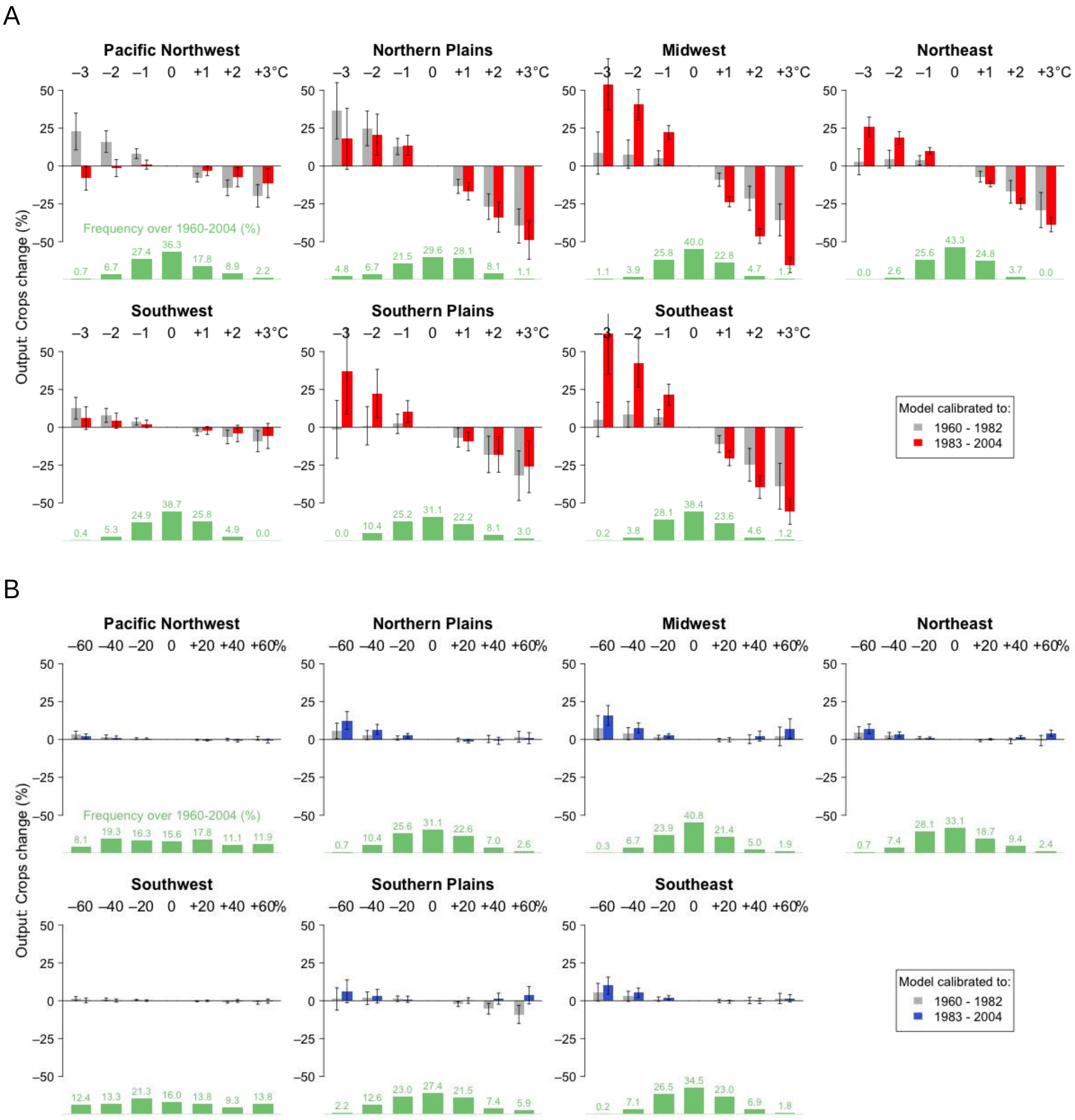

Fig. S14. Predicted crop output changes. Predicted crop output changes from summer temperature (**A**) and precipitation (**B**) change. The figure represents the same analysis shown in Fig. 3 and S5 but applied to crop output.

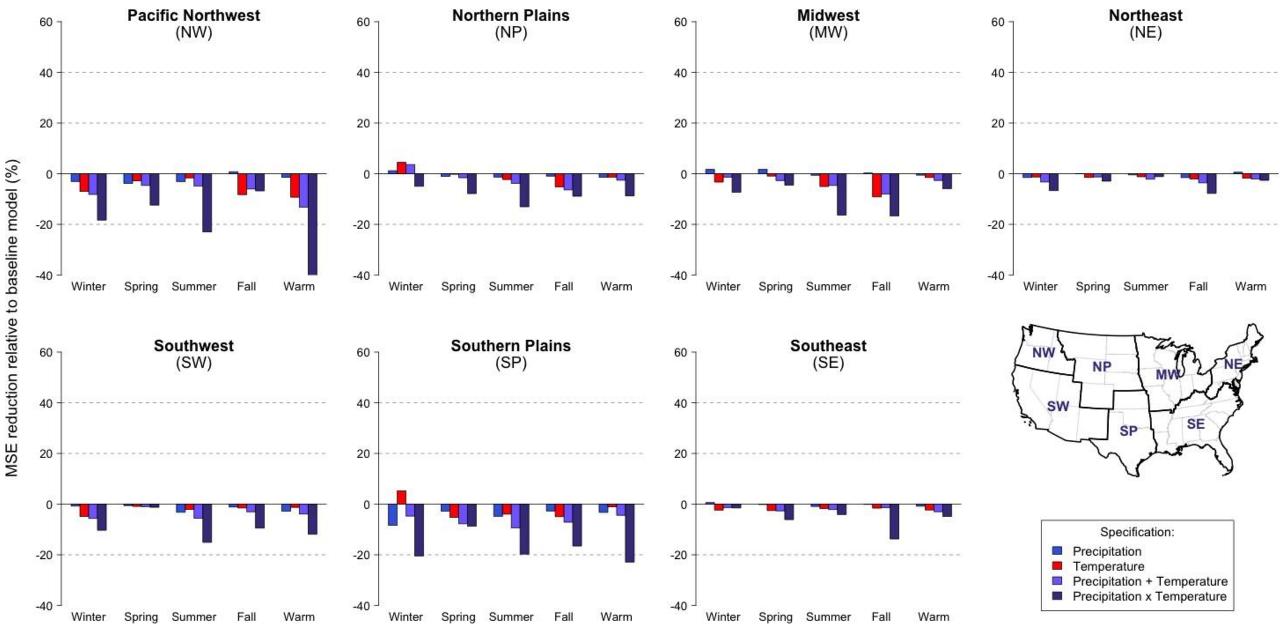

Fig. S15. Average reduction in MSE of out-of-sample predictions in livestock output relative to a model without weather variables (1960–2004). This figure is analogous to fig. S3 but applied to livestock output.

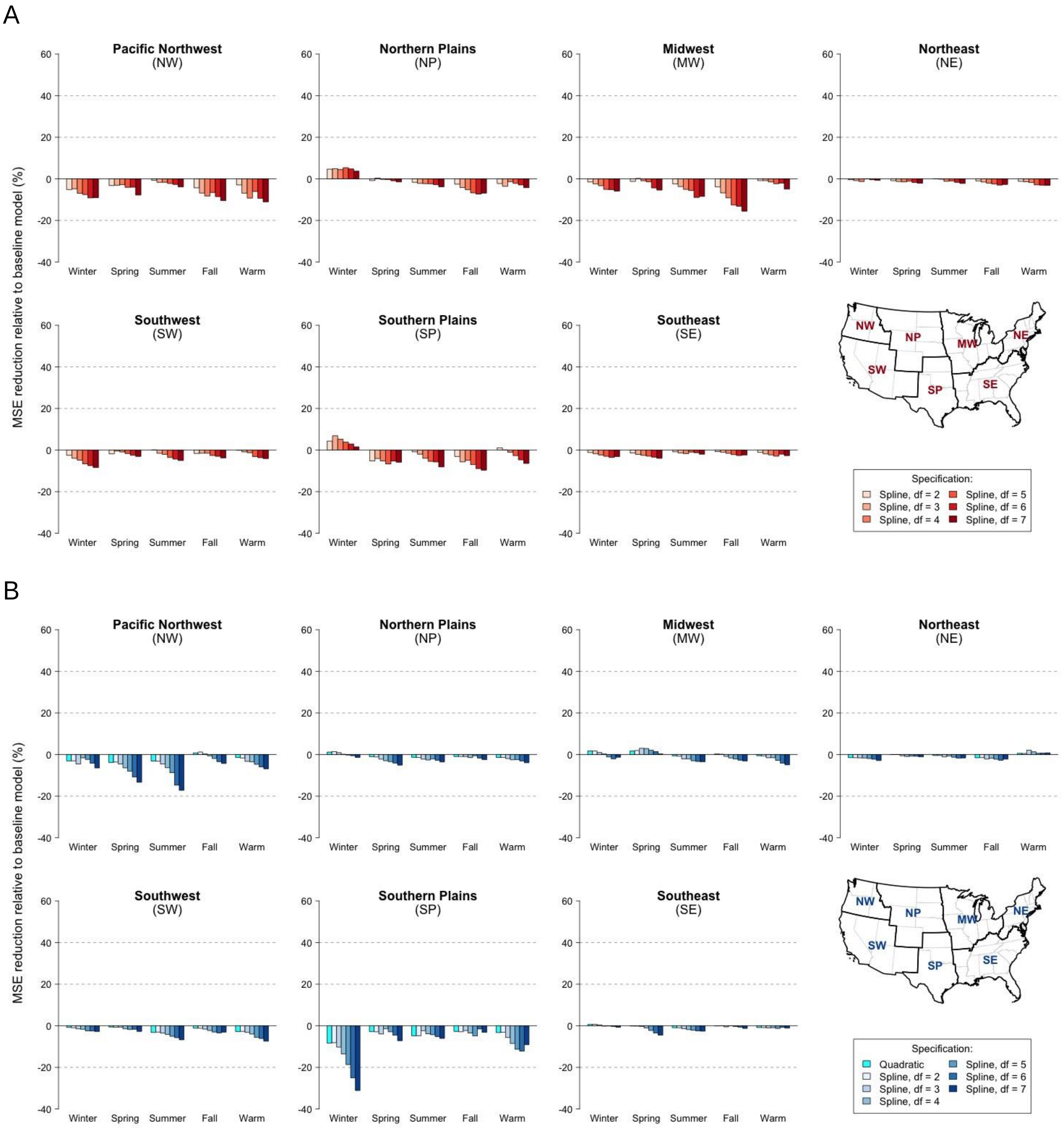

Fig. S16. Crop output predictability under varying flexibilities for temperature and precipitation variables. Average reduction in Mean Squared Error (MSE) of out-of-sample predictions in Livestock Output relative to a model without weather variables (1960-2004) for varying levels of flexibility of (A) temperature and (B) precip- itation response functions. This figure is analogous to fig. S4 but applied to livestock output.

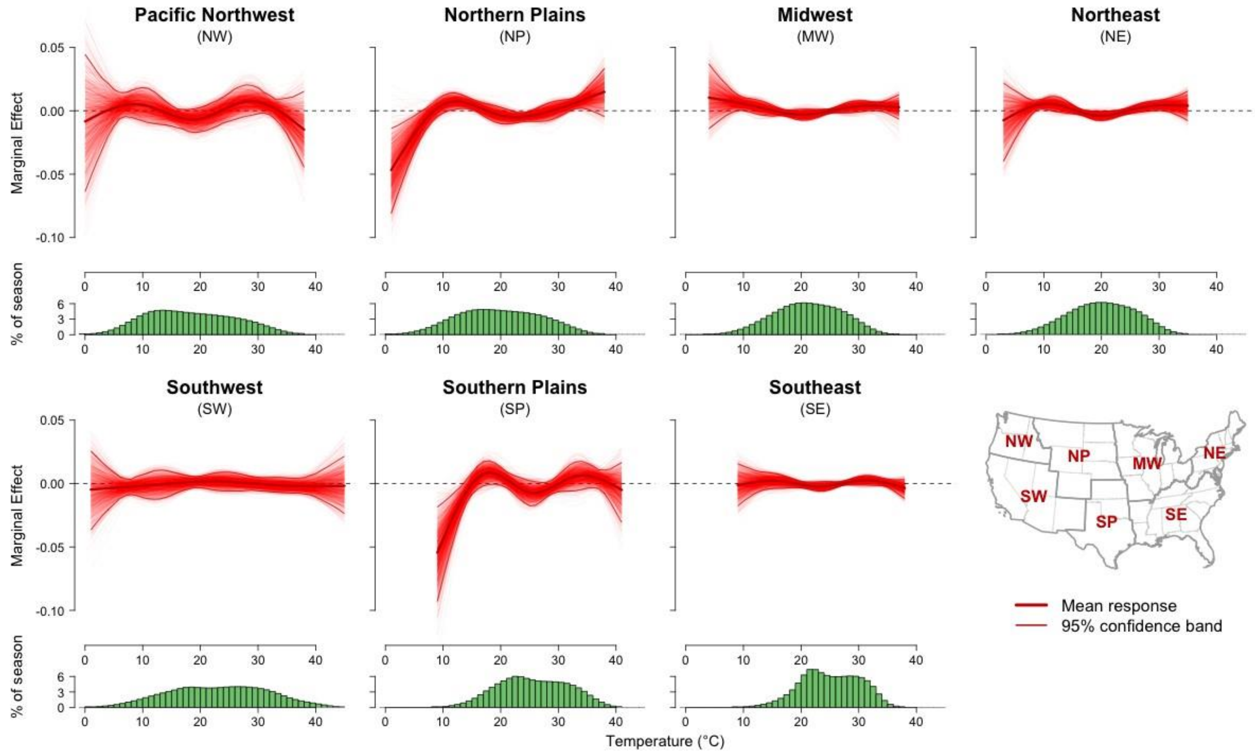

Fig. S17. Livestock output response to summer temperature by region. The overlay of gold response functions for each region corresponds to 1,000 response functions derived from bootstrapped regressions in which years of data (1960-2004) were sampled with replacement. Mean and 95% confidence intervals are represented in darker lines. Functions were estimated separately for each USDA climate hub region based on state-level crop output and summer (June-August) weather data. The green histograms below the response curves represent the percent of the time spent in each temperature bin during the summer.

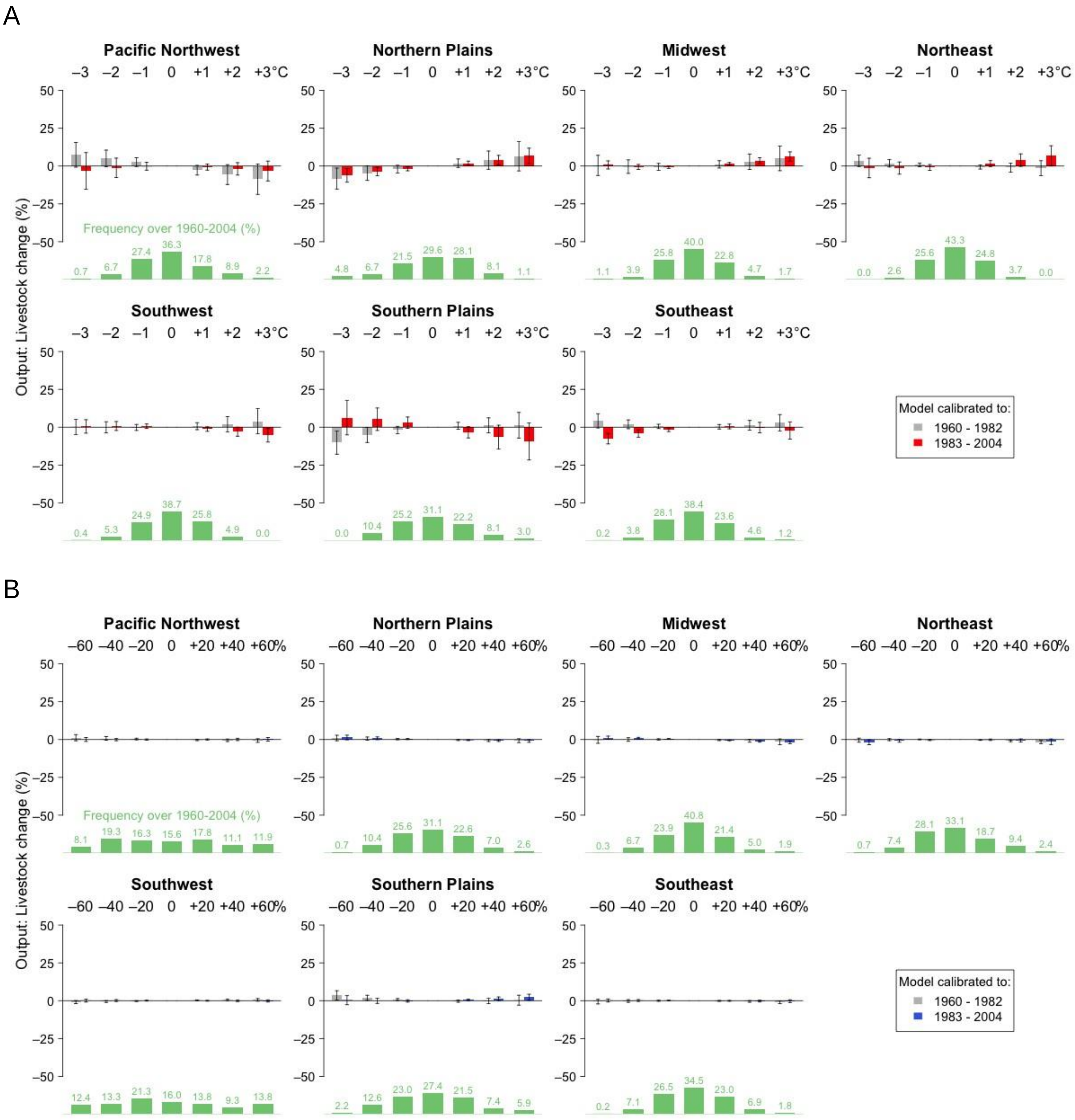

Fig. S18. Predicted livestock output changes. Predicted livestock output changes from summer temperature (**A**) and precipitation (B) change. The figure represents the same analysis shown in Fig. 3 and S5 but applied to livestock output.

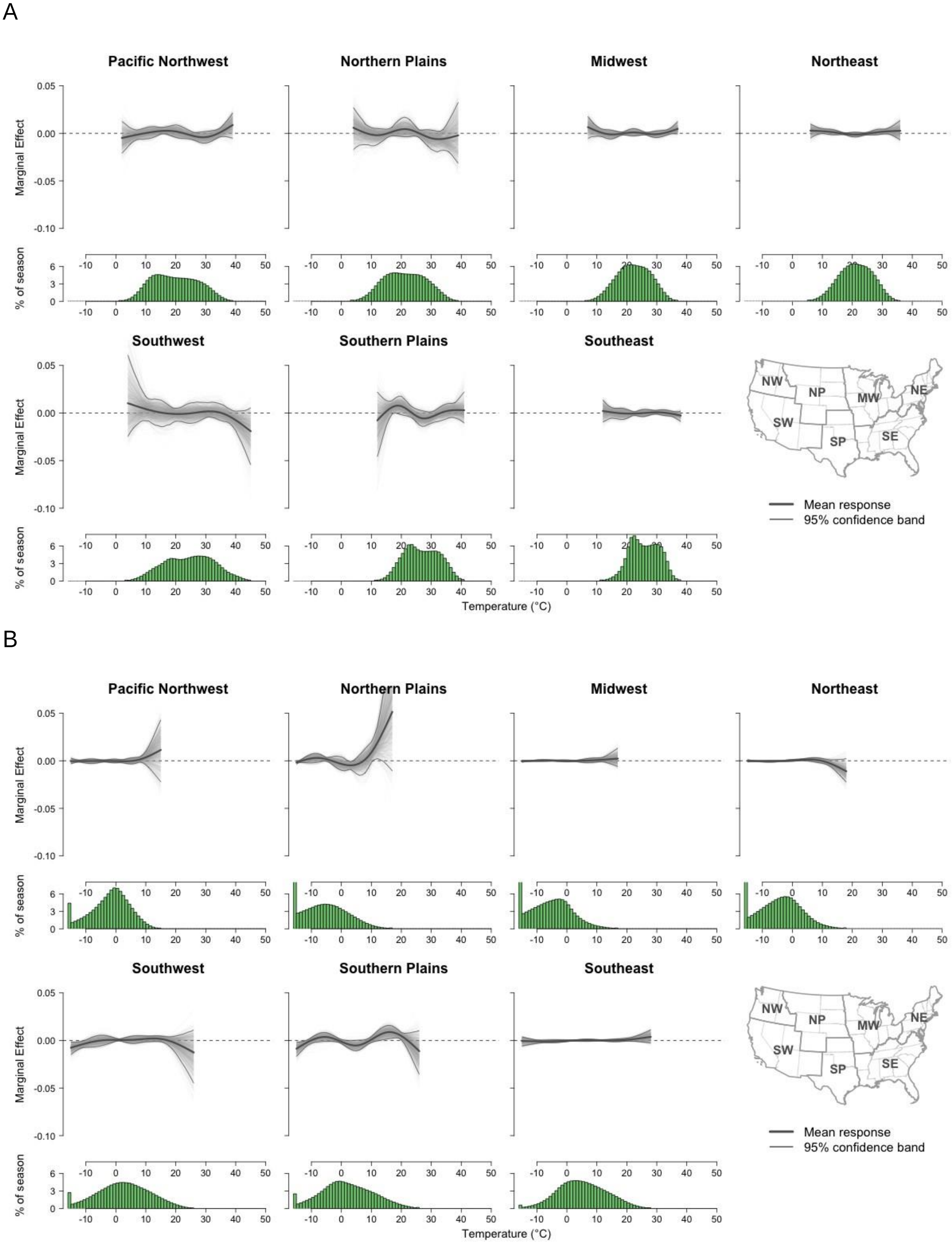

Fig. S19. Milk production per cow response to monthly temperature by region. These figures are analogous to Fig. 2 in the manuscript and figs. S12 and S17 in this appendix. The estimation here, however, is applied to monthly data for (A) July and (B) January.

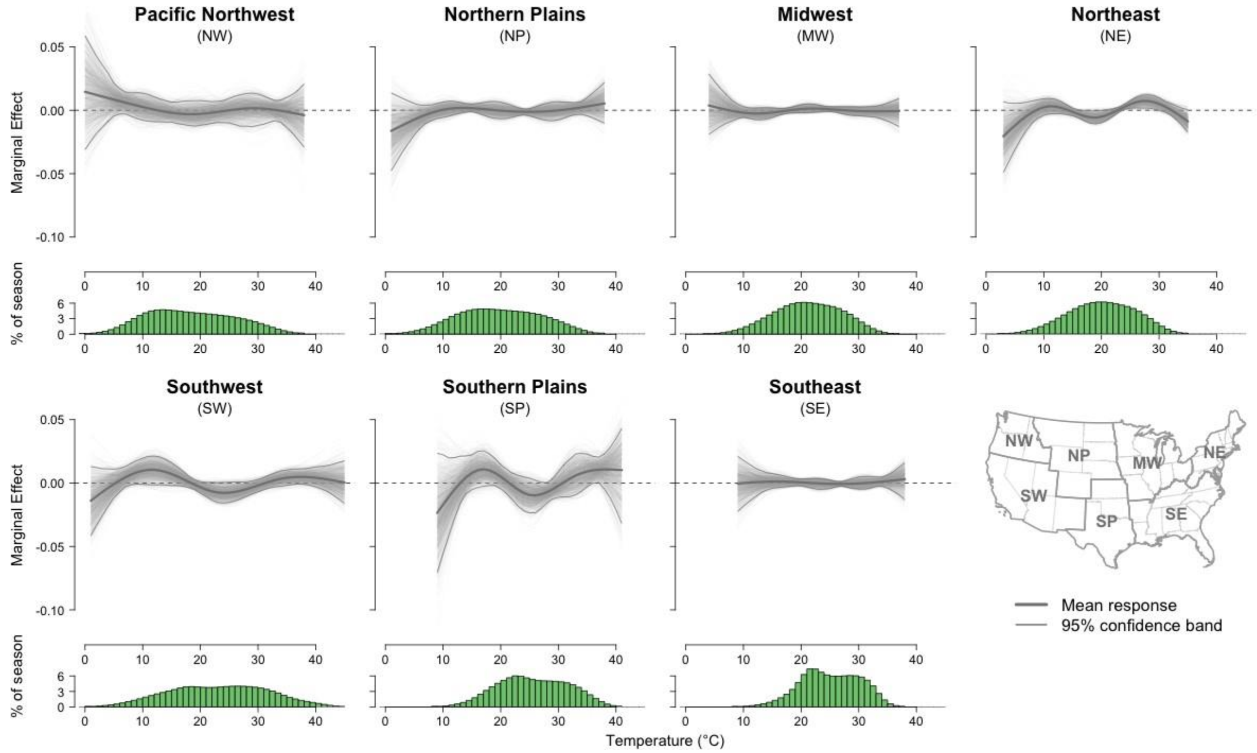

Fig. S20. Aggregate input response to summer temperature by region. This figure is analogous for Fig. 2 in the paper and figs. S12 and S17 in this appendix. The overlay of response functions for each region corresponds to 1,000 response functions derived from bootstrapped regressions in which years of data (1960-2004) were sampled with replacement. Mean and 95% confidence intervals are represented in darker lines. Functions were estimated separately for each USDA climate hub region based on state-level aggregate input and summer (June-August) weather data. The green histograms below the response curves represent the percent of the time spent in each temperature bin during the summer.

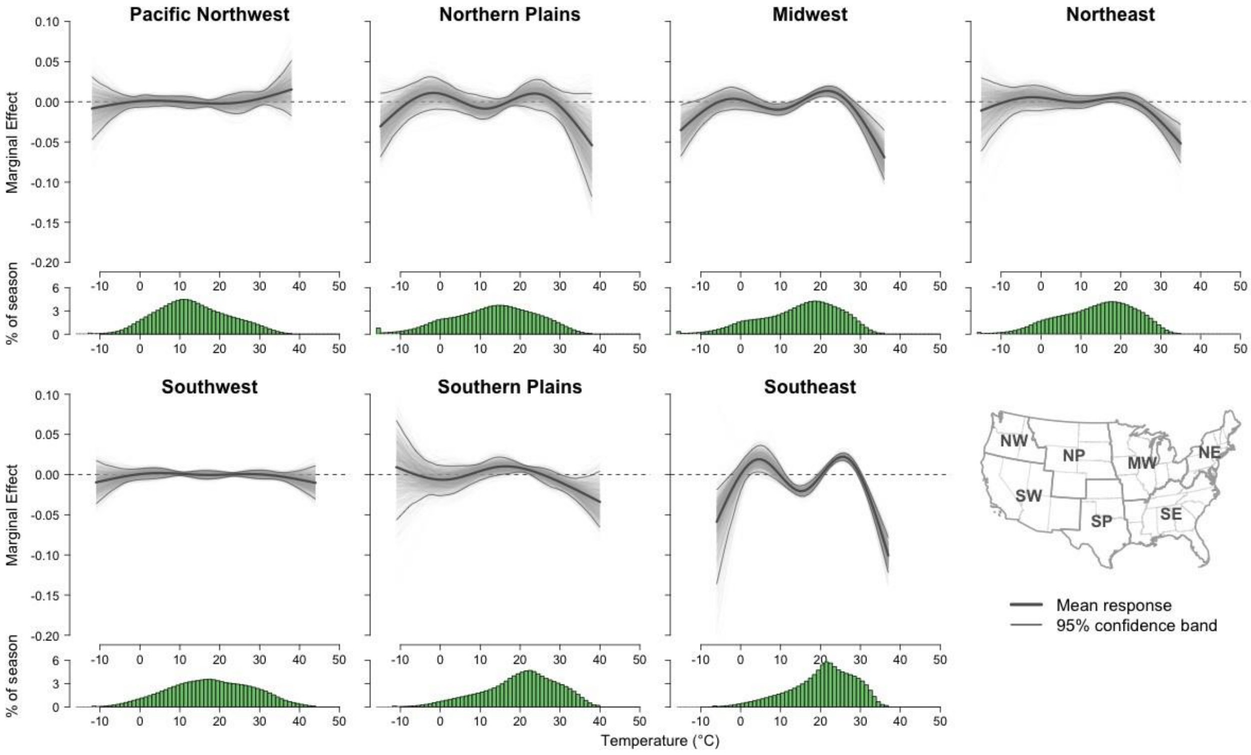

Fig. S21. Hay yield response to summer temperature by region. This figure is analogous for Fig. 2 in the paper and figs. S12 and S17 in this appendix. The overlay of response functions for each region corresponds to 1,000 response functions derived from bootstrapped regressions in which years of data (1960-2004) were sampled with replacement. Mean and 95% confidence intervals are represented in darker lines. Functions were estimated separately for each USDA climate hub region based on state-level hay yields and summer (June-August) weather data. The green histograms below the response curves represent the percent of the time spent in each temperature bin during the summer.

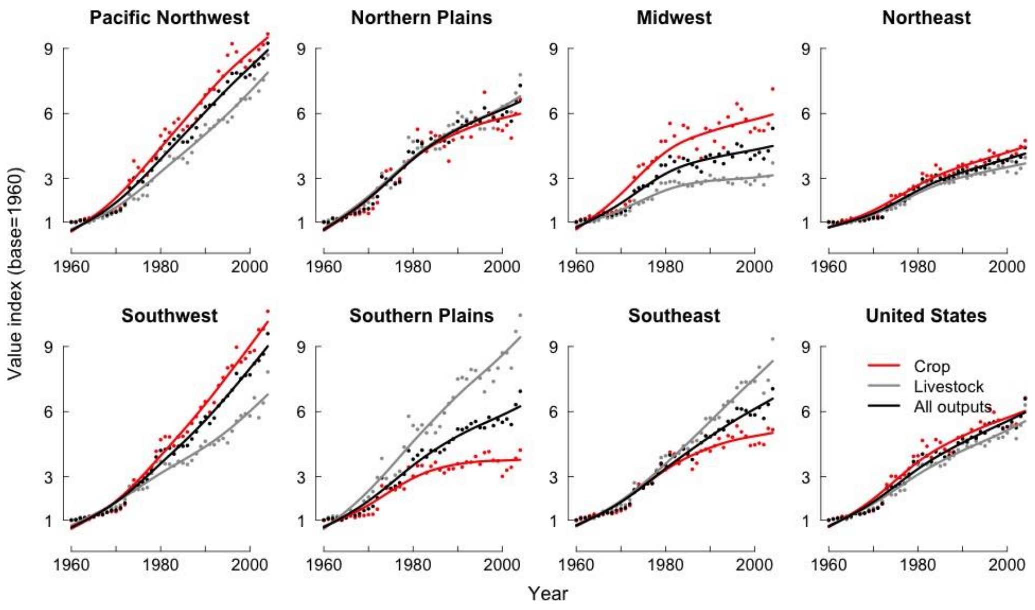

Fig. S22. Growth of regional production value. Each panel depicts the evolution of an index (base year=1960 for each region) representing the value of crops (red), livestock (grey) and total (black) production.

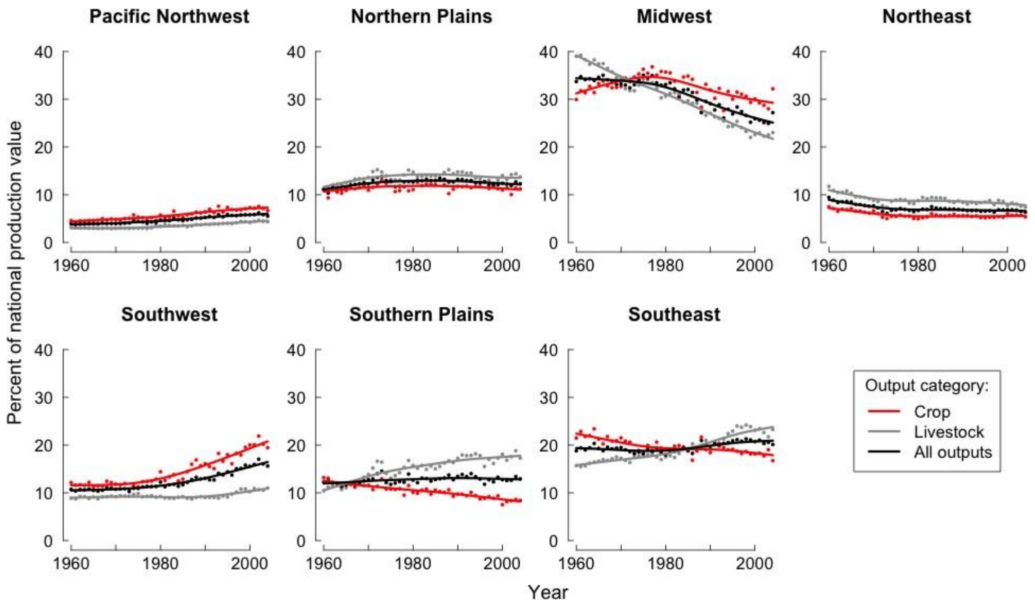

Fig. S23. Contribution to national production. Each panel represents the share that each climate hub region contributes to the national value in crops (red), livestock (grey) and total production (black).

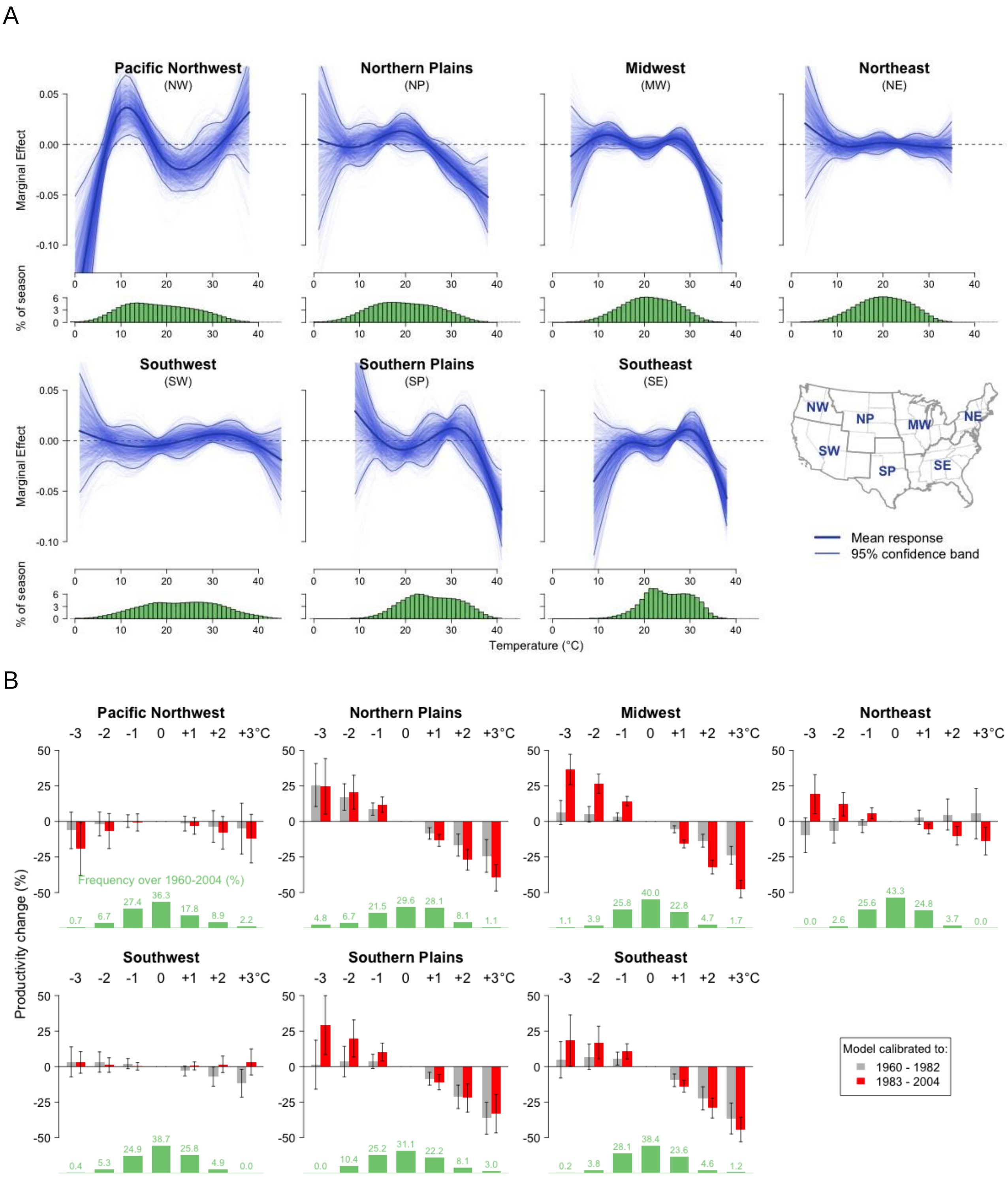

Fig. S24. Productivity response to summer temperature change based on a quadratic time trend. The model is identical to the preferred model in the manuscript but for the dependent variable (which is not de-trended prior to estimation) and the inclusion of the quadratic trend. (**A**) TFP response to summer temperature fluctuations. This is analogous to Fig. 2 in the manuscript. (**B**) Predicted changes in TFP from uniform changes of summer temperature distribution. This is analogous to Fig. 3 in the manuscript.

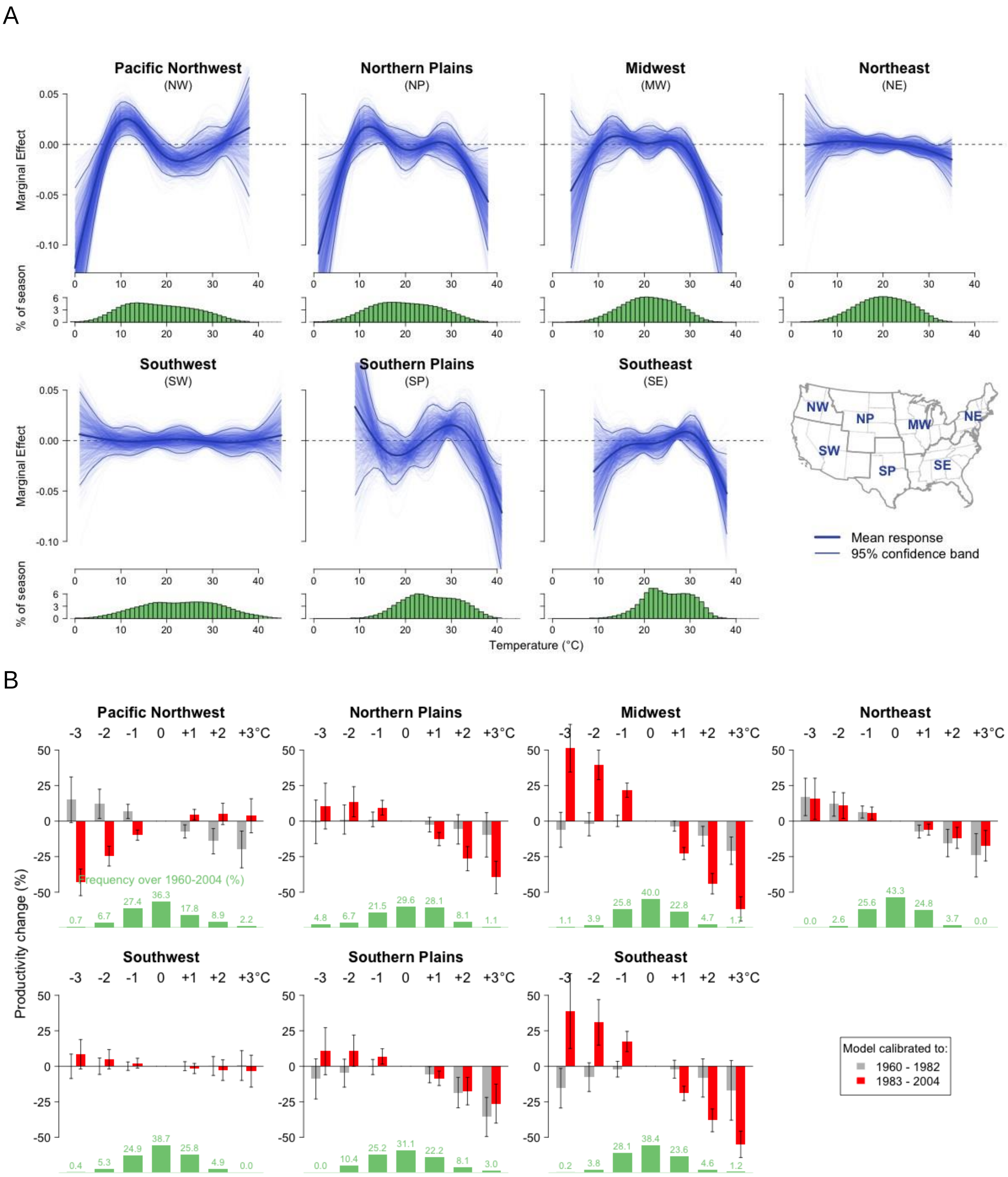

Fig. S25. Productivity response to summer temperature change based on first differences. The model is identical to the preferred model in the manuscript but for the dependent variable, which is de-trended by taking the first differences. (**A**) TFP response to summer temperature fluctuations. This is analogous to Fig. 2 in the manuscript. (**B**) Predicted changes in TFP from uniform changes of summer temperature distribution. This is analogous to Fig. 3 in the manuscript.

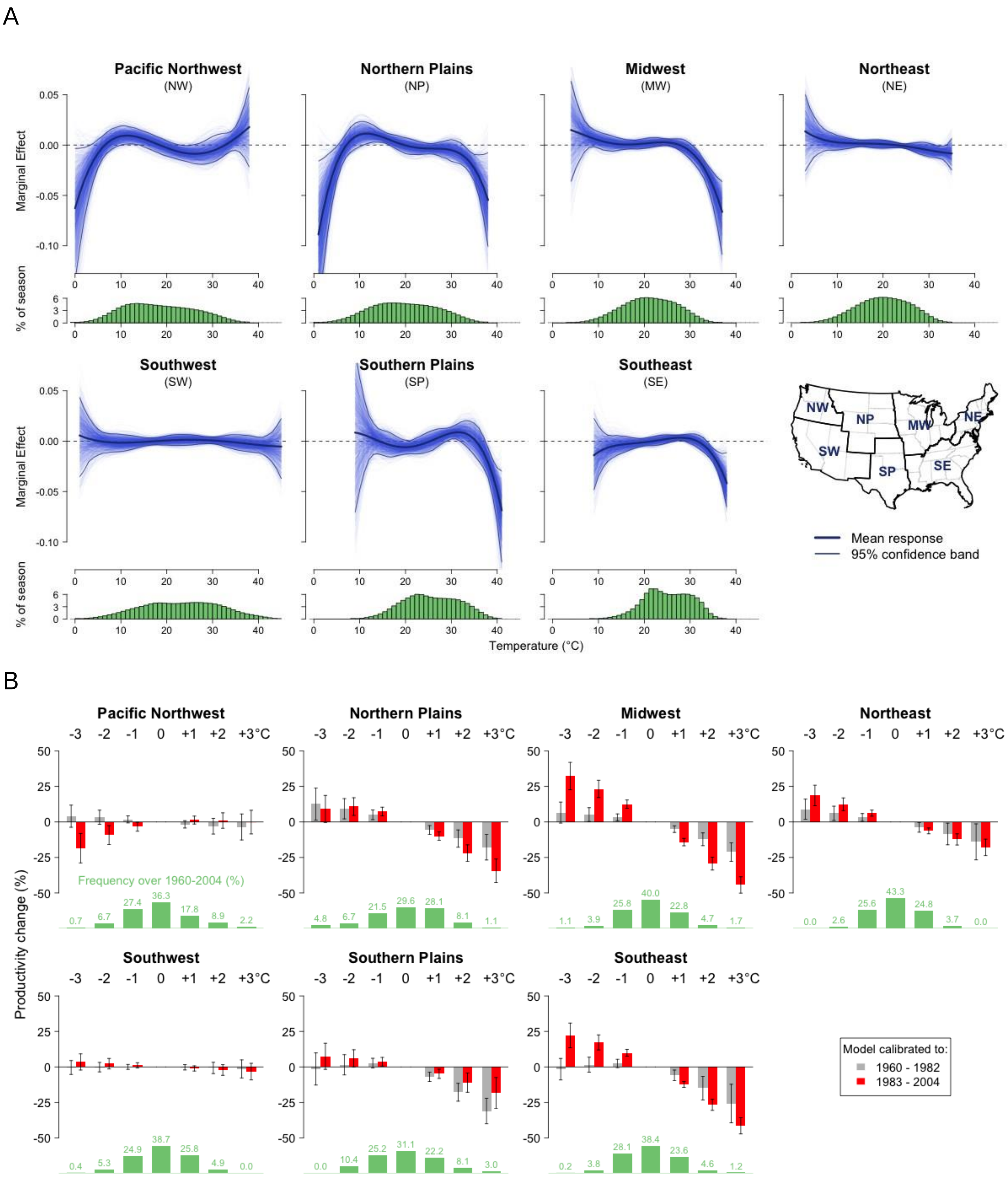

Fig. S26. Productivity response to summer temperature change based on a Chebyshev polynomial of degree 4. (**A**) TFP response to temperature fluctuations. This is analogous to Fig. 2 in the manuscript. (**B**) Predicted changes in TFP from uniform changes of summer temperature distribution. This is analogous to Fig. 3 in the manuscript.

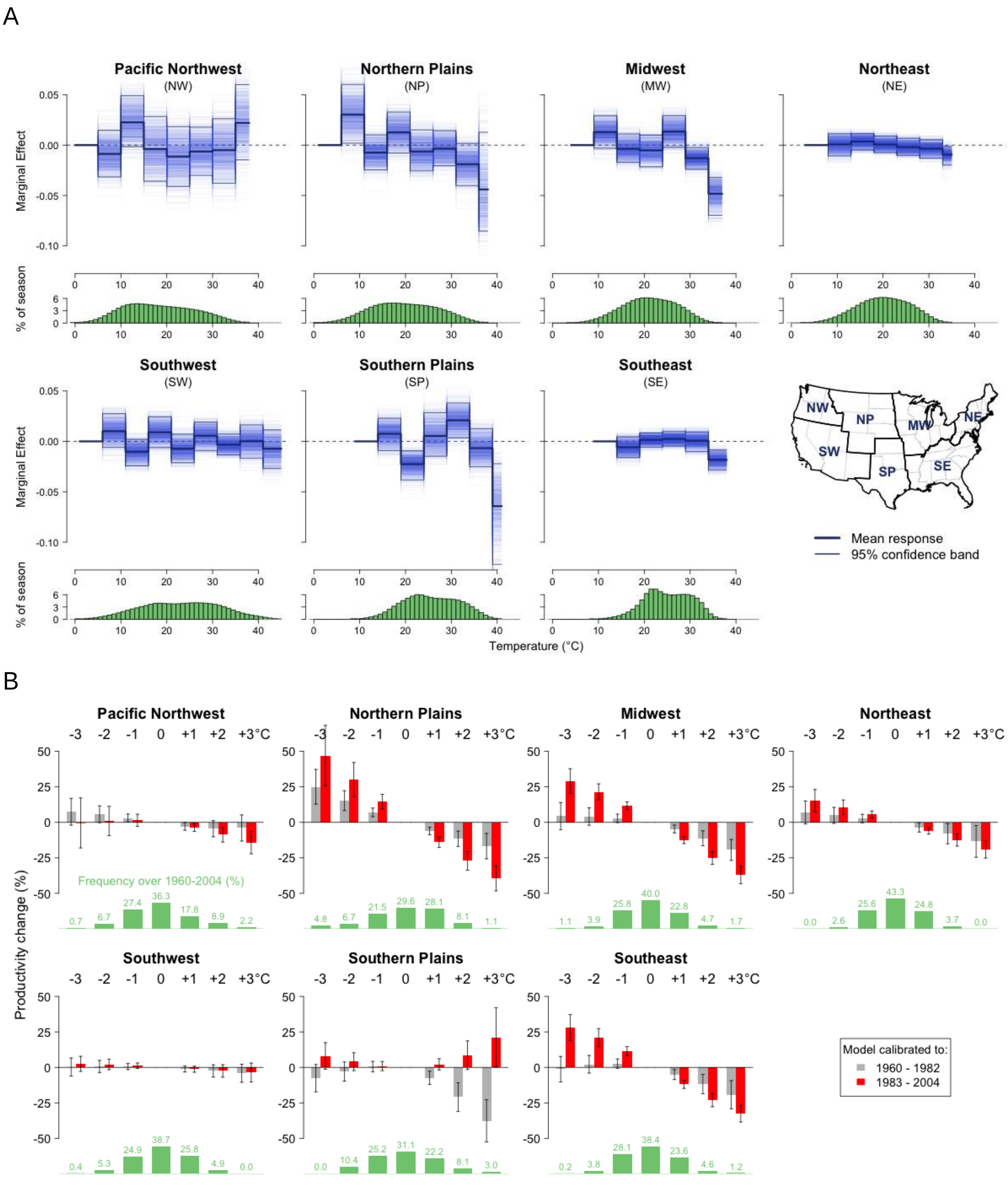

Fig. S27. Productivity response to summer temperature change based on a step function with 5°C steps. (**A**) TFP response to temperature fluctuations. This is analogous to Fig. 2 in the manuscript. (**B**) Predicted changes in TFP from uniform changes of summer temperature distribution. This is analogous to Fig. 3 in the manuscript.

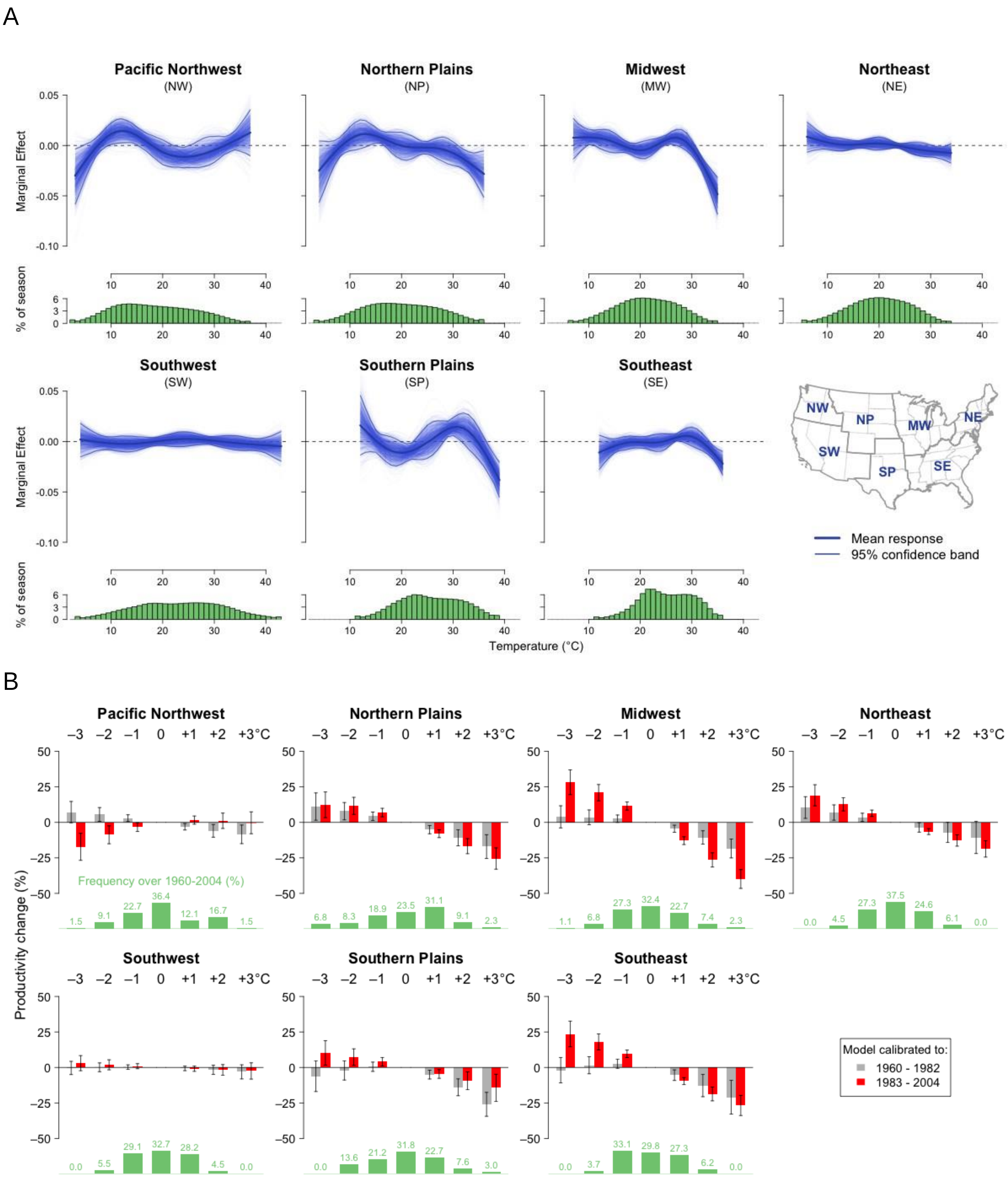

Fig. S28. Productivity response to summer temperature change based on a higher tail aggregation threshold of 0.5%. (A) TFP response to temperature fluctuations. This is analogous to Fig. 2 in the manuscript. (B) Predicted changes in TFP from uniform changes of summer temperature distribution. This is analogous to Fig. 3 in the manuscript.

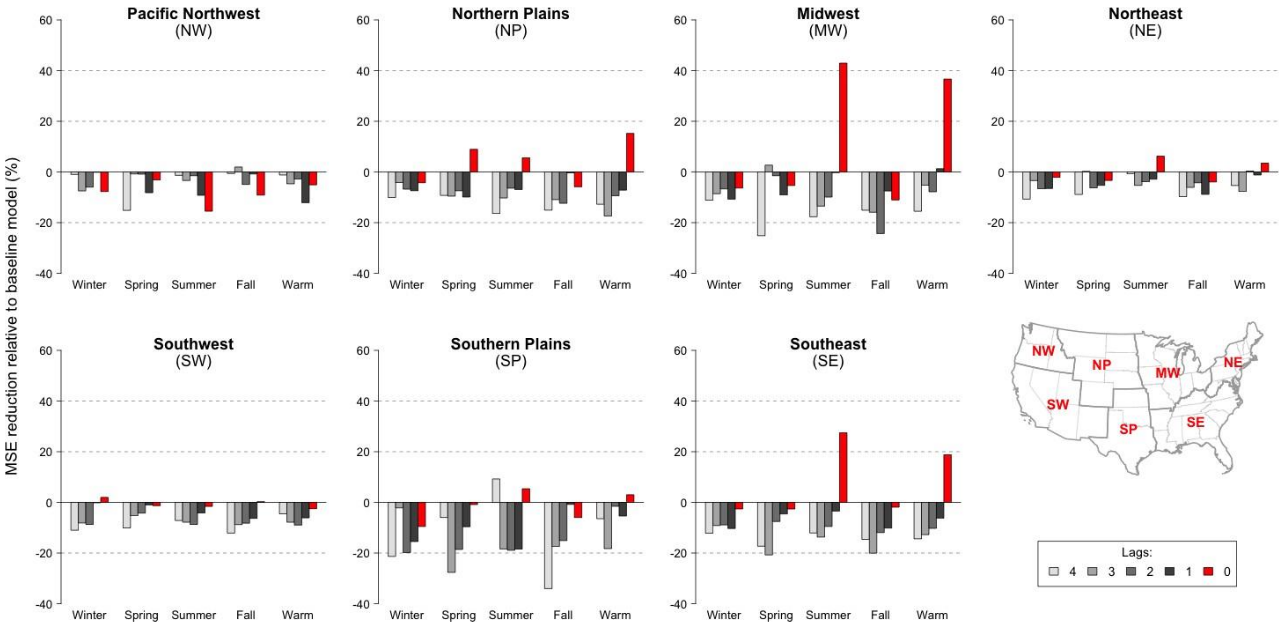

Fig. S29. Average reduction in MSE in TFP relative to a model without weather variables (1960–2004) for models with lagged weather variables. The model without lags (lag=0) depicted in red corresponds to the preferred model incorporating linear and quadratic terms for total precipitation as well as a natural spline with 4 degrees of freedom (3 knots) for temperature exposure for the relevant period. The lagged models depicted in grey correspond to the same model except that weather variables are lagged by [lag] number of years.

Table S1. Test results for climate parameter stability in TFP regressions. The table shows the results of Wald tests testing the stability of climate coefficients between 1960-1982 and 1983-2004. Models are estimated by USDA Climate Hub region and errors are clustered by year. The parameters tested include: "T and P" (temperature and precipitation), "T" (only temperature) and "P" (only precipitation). Climatic variables correspond to the summer. See Supplementary Fig. S2 for a clarification of regional acronyms.

Climate Hug Region	Parameters Tested	N	Chi sq.	p-value
NW	T and P	126	9.33	0.156
	T	124	4.17	0.384
	P	122	7.32	0.026
SW	T and P	214	2.69	0.846
	T	212	2.18	0.704
	P	210	0.30	0.861
NP	T and P	258	3.53	0.740
	T	256	2.37	0.669
	P	254	1.25	0.536
SP	T and P	126	7.27	0.296
	T	124	5.83	0.213
	P	122	2.78	0.249
MW	T and P	346	10.69	0.098
	T	344	10.54	0.032
	P	342	1.48	0.477
SE	T and P	478	2.37	0.883
	T	476	2.01	0.735
	P	474	0.17	0.921
NE	T and P	522	7.33	0.292
	T	520	1.24	0.872
	P	518	6.05	0.049

Table S2. Test results for climate parameter stability in TFP regressions excluding 1983. This is similar to Tab. S1 but excluding year 1983 to avoid confounding the Payments-in-Kind program and the 1983 drought. The table shows the results of Wald tests testing the stability of climate coefficients between 1960-1982 and 1984-2004. Models are estimated by USDA Climate Hub region and errors are clus- tered by year. The parameters tested include: "T and P" (temperature and precipitation), "T" (only temperature) and "P" (only precipitation). Climatic variables correspond to the summer.

Climate Hug Region	Parameters Tested	N	Chi sq.	p-value
NW	T and P	123	9.00	0.174
	T	121	5.16	0.271
	P	119	7.41	0.025
SW	T and P	209	0.74	0.994
	T	207	0.57	0.966
	P	205	0.11	0.945
NP	T and P	252	2.41	0.879
	T	250	1.79	0.774
	P	248	1.34	0.512
SP	T and P	123	6.88	0.332
	T	121	6.34	0.175
	P	119	2.88	0.237
MW	T and P	338	10.80	0.095
	T	336	9.08	0.059
	P	334	1.16	0.560
SE	T and P	467	2.99	0.810
	T	465	2.48	0.648
	P	463	0.30	0.861
NE	T and P	510	8.63	0.195
	T	508	1.57	0.814
	P	506	5.98	0.050

Table S3. Estimates and *P* values for test of differences in impact on TFP of varying temperature scenarios. Mean impacts and standard deviations are computed based on bootstrapped models calibrated for two different periods (1960-1982 and 1983-2004).

Climate Hub Region		Change in Summer Temperature (°C)					
		−3	−2	−1	+1	+2	+3
NW	Impact (%, 1960-1982)	5.3	4.6	2.7	-2.9	-5.6	-7.9
	Impact (%, 1983-2004)	-16.9	-8.1	-2.7	0.6	-0.2	-1.2
	P-value	0.088	0.122	0.165	0.268	0.310	0.336
SW	Impact (%, 1960-1982)	-0.6	-0.1	0.1	-0.4	-1.1	-2.2
	Impact (%, 1983-2004)	4.4	2.8	1.3	-1.3	-2.6	-3.9
	P-value	0.320	0.335	0.350	0.374	0.383	0.390
NP	Impact (%, 1960-1982)	9.2	6.8	3.7	-4.4	-9.2	-14.4
	Impact (%, 1983-2004)	11.4	11.8	7.5	-9.4	-19.7	-29.8
	P-value	0.395	0.347	0.286	0.211	0.196	0.195
SP	Impact (%, 1960-1982)	-3.1	0.1	1.6	-5.6	-15.0	-27.2
	Impact (%, 1983-2004)	12.6	9.3	5.1	-5.9	-12.4	-19.2
	P-value	0.211	0.238	0.299	0.398	0.383	0.337
MW	Impact (%, 1960-1982)	5.8	4.6	2.9	-4.7	-11.3	-19.7
	Impact (%, 1983-2004)	31.3	23.0	12.6	-14.2	-29.3	-44.2
	P-value	0.032	0.018	0.011	0.005	0.004	0.004
SE	Impact (%, 1960-1982)	-0.7	2.0	2.5	-5.5	-13.7	-23.6
	Impact (%, 1983-2004)	23.3	17.8	9.8	-11.0	-22.8	-34.6
	P-value	0.053	0.049	0.066	0.172	0.235	0.285
NE	Impact (%, 1960-1982)	10.1	6.6	3.4	-3.6	-7.5	-11.6
	Impact (%, 1983-2004)	19.8	13.0	6.5	-6.4	-12.5	-18.4
	P-value	0.262	0.261	0.276	0.315	0.338	0.357

Table S4. Test results for climate parameter stability in crop output regressions. The table shows the results of Wald tests testing the stability of climate coefficients between 1960-1982 and 1983-2004. Models are estimated by USDA Climate Hub region and errors are clustered by year. The parameters tested include: "T and P" (temperature and precipitation), "T" (only temperature) and "P" (only precipitation). Climatic variables correspond to the summer.

**Climate Hug Region**	**Parameters Tested**	**N**	**Chi sq.**	**p-value**
NW	T and P	125	7.19	0.304
	T	123	4.21	0.378
	P	121	6.06	0.048
SW	T and P	213	2.18	0.902
	T	211	0.82	0.936
	P	209	0.44	0.802
NP	T and P	257	2.51	0.867
	T	255	0.63	0.960
	P	253	0.75	0.688
SP	T and P	125	13.60	0.034
	T	123	3.85	0.426
	P	121	3.69	0.158
MW	T and P	345	9.81	0.133
	T	343	9.43	0.051
	P	341	4.97	0.084
SE	T and P	477	12.00	0.062
	T	475	9.89	0.042
	P	473	2.08	0.354
NE	T and P	521	7.05	0.317
	T	519	2.75	0.600
	P	517	3.21	0.201

Table S5. Estimates and *P* values for test of differences in impact on crop output of varying temperature scenarios. Mean impacts and standard deviations are computed based on bootstrapped models calibrated for two different periods (1960-1982 and 1983-2004).

Climate Hub Region		Change in Summer Temperature (°C)					
		−3	−2	−1	+1	+2	+3
NW	Impact (%, 1960-1982)	22.8	16.2	8.2	-7.7	-14.4	-19.7
	Impact (%, 1983-2004)	-7.9	-1.3	1.0	-3.1	-7.3	-11.3
	P-value	0.042	0.064	0.101	0.220	0.275	0.314
SW	Impact (%, 1960-1982)	12.6	7.8	3.7	-3.3	-6.4	-9.2
	Impact (%, 1983-2004)	6.0	4.3	2.2	-2.1	-4.1	-5.8
	P-value	0.326	0.348	0.362	0.375	0.379	0.380
NP	Impact (%, 1960-1982)	36.5	24.9	12.9	-13.4	-26.8	-39.6
	Impact (%, 1983-2004)	18.0	20.9	13.8	-16.8	-33.8	-48.8
	P-value	0.318	0.389	0.397	0.358	0.345	0.345
SP	Impact (%, 1960-1982)	-1.4	1.0	2.2	-6.8	-18.0	-32.0
	Impact (%, 1983-2004)	36.9	22.2	10.4	-9.4	-18.1	-26.2
	P-value	0.212	0.232	0.279	0.382	0.399	0.387
MW	Impact (%, 1960-1982)	8.6	7.9	5.4	-9.0	-21.3	-35.6
	Impact (%, 1983-2004)	54.1	40.6	22.2	-23.7	-46.3	-65.3
	P-value	0.046	0.023	0.014	0.009	0.011	0.016
SE	Impact (%, 1960-1982)	5.1	8.4	6.8	-11.1	-24.9	-39.1
	Impact (%, 1983-2004)	62.0	42.5	21.5	-20.7	-39.6	-55.8
	P-value	0.059	0.068	0.089	0.169	0.213	0.252
NE	Impact (%, 1960-1982)	2.9	4.6	3.8	-7.0	-17.0	-29.1
	Impact (%, 1983-2004)	25.9	18.6	10.1	-11.8	-24.9	-38.6
	P-value	0.041	0.061	0.095	0.196	0.253	0.298

Table S6. Test results for climate parameter stability in livestock output regressions. The table shows the results of Wald tests testing the stability of climate coefficients between 1960-1982 and 1983-2004. Models are estimated by USDA Climate Hub region and errors are clustered by year. The parameters tested include: "T and P" (temperature and precipitation), "T" (only temperature) and "P" (only precipitation). Climatic variables correspond to the summer.

Climate Hug Region	Parameters Tested	N	Chi sq.	p-value
NW	T and P	125	2.69	0.846
	T	123	0.67	0.956
	P	121	0.19	0.911
SW	T and P	213	4.66	0.588
	T	211	3.98	0.409
	P	209	1.43	0.489
NP	T and P	257	3.61	0.729
	T	255	2.39	0.664
	P	253	0.03	0.983
SP	T and P	125	1.92	0.927
	T	123	1.51	0.825
	P	121	1.07	0.586
MW	T and P	345	2.42	0.877
	T	343	1.15	0.887
	P	341	1.83	0.401
SE	T and P	477	11.24	0.081
	T	475	8.67	0.070
	P	473	1.39	0.499
NE	T and P	521	12.01	0.062
	T	519	4.43	0.351
	P	517	4.38	0.112

Table S7. Estimates and *P* values for test of differences in impact on livestock output of varying temperature scenarios. Mean impacts and standard deviations are computed based on bootstrapped models calibrated for two different periods (1960-1982 and 1983-2004).

Climate Hub Region		Change in Summer Temperature (°C)					
		−3	−2	−1	+1	+2	+3
NW	Impact (%, 1960-1982)	7.5	5.0	2.6	-2.8	-5.7	-8.7
	Impact (%, 1983-2004)	-3.2	-1.2	-0.1	-0.6	-1.8	-3.3
	P-value	0.306	0.305	0.313	0.341	0.353	0.360
SW	Impact (%, 1960-1982)	0.2	0.0	-0.2	0.7	1.9	4.0
	Impact (%, 1983-2004)	0.5	0.8	0.7	-1.2	-3.0	-5.3
	P-value	0.398	0.392	0.376	0.314	0.278	0.243
NP	Impact (%, 1960-1982)	-8.3	-4.6	-2.0	1.9	3.9	6.5
	Impact (%, 1983-2004)	-6.1	-3.7	-1.8	1.9	4.0	6.8
	P-value	0.385	0.393	0.397	0.399	0.399	0.399
SP	Impact (%, 1960-1982)	-10.2	-5.2	-1.9	0.9	1.2	1.3
	Impact (%, 1983-2004)	6.3	5.3	3.0	-3.3	-6.5	-9.4
	P-value	0.192	0.202	0.216	0.255	0.281	0.308
MW	Impact (%, 1960-1982)	0.4	-0.3	-0.5	1.1	2.9	5.1
	Impact (%, 1983-2004)	0.8	-0.6	-0.8	1.5	3.7	6.2
	P-value	0.398	0.398	0.396	0.395	0.395	0.396
SE	Impact (%, 1960-1982)	4.2	1.9	0.5	0.3	1.3	2.9
	Impact (%, 1983-2004)	-7.6	-4.2	-1.6	0.5	-0.3	-2.2
	P-value	0.051	0.104	0.208	0.397	0.379	0.323
NE	Impact (%, 1960-1982)	3.5	1.8	0.8	-0.6	-1.0	-1.4
	Impact (%, 1983-2004)	-1.3	-1.3	-0.9	1.7	3.9	6.7
	P-value	0.326	0.318	0.304	0.260	0.250	0.247

Table S8. Decomposition of production costs by livestock output category, farm resource region, and over time (in %). Source: ERS Commodity Costs and Returns: https://www.ers.usda.gov/data-products/commodity-costs-and-returns/ (accessed 7/27/2017)

	Hogs			Milk			Cow-Calf	
	1998 -2003	2004 -2008	2009 -2016	2000 -2004	2005 -2009	2010 -2016	1996 -2007	2008 -2016
**United States**								
Operating costs	67.1	75.3	81.1	52.0	62.9	68.2	50.8	44.3
→ Feed	35.4	38.2	47.5	37.0	47.3	55.8	30.9	30.7
→ Other	31.7	37.2	33.5	15.0	15.5	12.4	19.9	13.6
–→ Fuel, lube and electricity	2.1	2.4	2.0	2.6	3.1	2.8	3.0	3.1
Allocated overhead	32.9	24.7	18.9	48.0	37.1	31.8	49.2	55.7
**Southern Seaboard**								
Operating costs	76.7	84.1	84.3	56.5	62.4	61.5		41.2
→ Feed	33.3	37.9	44.6	38.9	46.4	48.8		33.8
→ Other	43.4	46.2	39.6	17.6	16.0	12.7		22.7
–→ Fuel, lube and electricity	1.6	2.0	1.6	2.8	3.3	3.5		11.1
Allocated overhead	23.3	15.9	15.7	43.5	37.6	38.5		3.2
**Prairie Gateway**								
Operating costs	65.3	69.6	71.0	75.8	74.2	74.9	55.3	45.0
→ Feed	34.4	42.0	35.0	56.4	58.3	62.2	29.3	28.3
→ Other	30.8	27.7	36.0	19.4	15.9	12.7	26.0	16.7
–→ Fuel, lube and electricity	1.8	3.2	3.3	2.6	3.1	2.2	3.7	3.0
Allocated overhead	34.7	30.4	29.0	24.2	25.8	25.1	44.7	55.0
**Northern Crescent**								
Operating costs	60.4	71.5	74.3	44.4	58.3	63.9		
→ Feed	35.0	43.5	47.1	29.5	41.9	51.1		
→ Other	25.4	28.0	27.2	14.9	16.4	12.8		
–→ Fuel, lube and electricity	2.4	2.4	2.2	2.7	3.3	2.8		
Allocated overhead	39.6	28.5	25.7	55.6	41.7	36.1		
**Heartland**								
Operating costs	65.0	75.4	82.8	47.8	57.9	63.2	46.5	51.0
→ Feed	36.7	37.8	49.5	34.8	41.3	50.0	31.1	35.7
→ Other	28.2	37.7	33.3	13.0	16.6	13.2	15.4	15.3
–→ Fuel, lube and electricity	2.3	2.3	1.9	2.3	3.0	3.1	2.2	2.7
Allocated overhead	35.0	24.6	17.2	52.2	42.1	36.8	53.5	49.0

Table S8: (continued)

	Hogs			Milk			Cow-Calf	
	1998 -2003	2004 -2008	2009 -2016	2000 -2004	2005 -2009	2010 -2016	1996 -2007	2008 -2016
**Eastern Uplands**								
Operating costs	56.6	59.0	74.7	51.2	51.5	57.5	52.0	39.8
→ Feed	32.8	29.6	55.2	38.9	38.3	46.5	35.3	29.3
→ Other	23.7	29.4	19.5	12.3	13.2	11.0	16.7	10.5
–→ Fuel, lube and electricity	2.9	3.8	3.1	2.1	2.7	2.9	2.5	2.7
Allocated overhead	43.4	41.0	25.3	48.8	48.5	42.5	48.0	60.2
**Fruitful Rim**								
Operating costs				64.8	71.3	76.4	51.8	40.4
→ Feed				48.8	57.1	64.7	29.2	26.8
→ Other				16.0	14.2	11.7	22.6	13.5
–→ Fuel, lube and electricity				2.8	2.9	2.6	3.2	3.0
Allocated overhead				35.2	28.7	23.6	48.2	59.6
**Northern Great Plains**								
Operating costs	71.7						48.3	53.2
→ Feed	32.1						33.1	40.8
→ Other	39.6						15.2	12.4
–→ Fuel, lube and electricity	1.7						2.8	3.0
Allocated overhead	28.3						51.7	46.8
**Mississippi Portal**								
Operating costs	37.6						34.0	36.5
→ Feed	22.3						21.6	26.1
→ Other	15.3						12.3	10.4
–→ Fuel, lube and electricity	2.6						2.8	4.2
Allocated overhead	62.4						66.0	63.5
**Basin and Range**								
Operating costs							46.8	47.6
→ Feed							27.2	33.1
→ Other							19.6	14.5
–→ Fuel, lube and electricity							2.7	4.0
Allocated overhead							53.2	52.4
